# Ideal resuscitation pressure for uncontrolled hemorrhagic shock in different ages and sexes of rats

**DOI:** 10.1186/cc12888

**Published:** 2013-09-10

**Authors:** Tao Li, Yu Zhu, Kunlun Tian, Mingying Xue, Xiaoyong Peng, Dan Lan, Liangming Liu

**Affiliations:** 1 State Key Laboratory of Trauma, Burns and Combined Injury, Second Department of Research Institute of Surgery, Daping Hospital, Third Military Medical University, Chongqing 400042, P.R. China

## Abstract

**Introduction:**

Our previous studies demonstrated that 50-60 mmHg mean arterial blood pressure was the ideal target hypotension for uncontrolled hemorrhagic shock during the active hemorrhage in sexually mature rats. The ideal target resuscitation pressure for immature and older rats has not been determined.

**Methods:**

To elucidate this issue, using uncontrolled hemorrhagic-shock rats of different ages and sexes (6 weeks, 14 weeks and 1.5 years representing pre-adult, adult and older rats, respectively), the resuscitation effects of different target pressures (40, 50, 60, 70 and 80 mmHg) on uncontrolled hemorrhagic shock during active hemorrhage and the age and sex differences were observed.

**Results:**

Different target resuscitation pressures had different resuscitation outcomes for the same age and sex of rats. The optimal target resuscitation pressures for 6-week-old, 14-week-old and 1.5-year-old rats were 40 to 50 mmHg, 50 to 60 mmHg and 70 mmHg respectively. Ideal target resuscitation pressures were significantly superior to other resuscitation pressures in improving the hemodynamics, blood perfusion, organ function and animal survival of uncontrolled hemorrhagic-shock rats (*P *< 0.01). For same target resuscitation pressures, the beneficial effect on hemorrhagic shock had a significant age difference (*P *< 0.01) but no sex difference (*P *> 0.05). Different resuscitation pressures had no effect on coagulation function.

**Conclusion:**

Hemorrhagic-shock rats at different ages have different target resuscitation pressures during active hemorrhage. The ideal target resuscitation hypotension for 6-week-old, 14-week-old and 1.5-year-old rats was 40 to 50 mmHg, 50 to 60 mmHg and 70 mmHg, respectively. Their resuscitation effects have significant age difference but had no sex difference.

## Introduction

Traumatic hemorrhagic shock is often seen in civilian and military situations. It is the major cause of early death in injured soldiers, accounting for ≈50% of deaths of battle personnel [1]. Fluid resuscitation is the common and very important treatment for many types of circulatory shock, particularly for traumatic hemorrhagic shock, besides dressing, immobilization and hemostasis for early emergency treatment.

Many studies, including animal studies and clinical trials, suggest that over-resuscitation and under-resuscitation can increase mortality [2-4]. Permissive hypotension has been advocated as a better means to carry out field resuscitation of penetrating trauma, and has been shown to lower mortality in models of uncontrolled hemorrhagic shock in anesthetized pigs and rats when compared with resuscitation designed to normalize blood pressure (normotensive resuscitation)[5-8]. Studies have shown that 50 to 60 mmHg target mean artery pressure (MAP) is an ideal resuscitation pressure for uncontrolled hemorrhagic shock before bleeding has been controlled. Too low (40 mmHg) or too high (80 mmHg) target resuscitation pressure worsens the resuscitation effect [5,7-9]. However, most of these studies are limited to adult victims: no study has been carried out in the pre-adult period and older victims [10-12]. This situation interferes with the broad application of permissive hypotension in pre-adults and traumatic hemorrhagic shock in older patients. Ascertaining the ideal target resuscitation pressure for uncontrolled hemorrhagic shock in pre-adults and older victims of traumatic hemorrhagic shock is important.

Several research studies have reported that sex and age have important roles in the morbidity and mortality observed in cardiovascular diseases and trauma [13-15]. Different ages and sexes of human bodies have differences with respect to organ function, especially for cardiovascular function [16-18], which is an important factor that affects tissue perfusion. Hence, we hypothesized that different ages and sexes of hemorrhagic-shock victims may need different permissive hypotension during active hemorrhage. Older victims may need a higher permissive hypotension than younger victims because the former have lower regulatory ability with regard to cardiovascular function.

To elucidate this issue, using uncontrolled hemorrhagic-shock rats of different ages (6 weeks, 14 weeks and 1.5 years, representing immature, mature and older, respectively), the resuscitation effects of different target pressures (40, 50, 60, 70 and 80 mmHg) on uncontrolled hemorrhagic shock during active hemorrhage were observed.

## Materials and methods

### Ethical approval of the study protocol

The present study was approved by the Research Council and Animal Care and Use Committee of the Research Institute of Surgery, Daping Hospital, Third Military Medical University (Chongqing, China). The approval number for the animal and clinical studies was YYLL (2012)006. The protocol conformed to the Guide for the Care and Use of Laboratory Animals published by the US National Institutes of Health (NIH Publication, 8th Edition, 2011).

### Animal management

Sprague-Dawley (SD) rats aged 6 weeks (body weight: female, 114 ± 9.2g; male, 115 ± 19.9 g), 14 weeks (body weight: female, 209 ± 9.3 g; male, 230 ± 14.9 g) or 1.5 years (body weight: female, 388 ± 39.7 g; male, 457 ± 59.2 g) were fasted for 12 hours but allowed water with sugar-salt solution (5% glucose saline) *ad libitum *before the experiment. On the day of experiment, rats were first anesthetized with sodium pentobarbital (30 mg/kg, intraperitoneally), which was added until the rats had no response to a needle stimulus of the front toe. The total amount of sodium pentobarbital was ~50 mg/kg. No rats presented apnea under this anesthesia.

The right femoral artery and the left and right femoral veins were catheterized with a polyethylene catheter (outer diameter, 0.96 mm; inner diameter,0.58 mm) to monitor the MAP with a Polygraph Physiological Recorder (SP844, Power Laboratory; AD Instruments, Castle Hill, NSW, Australia), bleeding and infusion. Left ventricular catheterization via the right carotid artery was performed for observation of hemodynamics. To prevent clot formation, the artery catheter was filled with normal (0.9%) saline containing 30 U/ml heparin. To maintain the body temperature at 37°C (monitored by anal thermometer), rats were placed on a warming plate. An uncontrolled hemorrhagic-shock model was reproduced as described previously by our research team [7,8]. The detailed operation procedures, including laparotomy, splenic parenchyma and splenic artery transaction, and the management of abdomen incision during the period of experiments are presented in Additional file 1. The animal supplier and their breeding conditions and food formula are also presented in Additional file 1

### Experimental phases

Experiments were classified into four phases. Phase I was the uncontrolled hemorrhagic shock (model stage). Blood was allowed to hemorrhage freely into the abdominal cavity. When the MAP decreased to 40 mmHg, this phase was achieved; it took 20 to 30 minutes to reach 40 mmHg.

Phase II was the resuscitation period before active bleeding was stopped in which rats were resuscitated at different target MAPs (40, 50, 60, 70 and 80 mmHg) for 1 hour with infusion of a mixture of 6% hydroxyethyl starch-130+ lactated Ringer's solution at a ratio of 1:2 [7,8].

Phase III was the definitive resuscitation period; after bleeding was stopped by full ligation of the splenic artery and vein, rats received whole blood and lactated Ringer's solution at a ratio of 1:2 to maintain the MAP at 80 to 90 mmHg for 2 hours. Whole blood and lactated Ringer's solution were infused simultaneously via left and right femoral venous catheters at a 1:2 infusion rate with two pumps (8714843, perfusor compact S; B. Braun Melsungen, Germany), and the infusion rate was regulated to maintain a stable target MAP. Whole blood was acquired from donor normal rats (one donor rat for two or three hemorrhagic shock rats).

**Figure 1:**
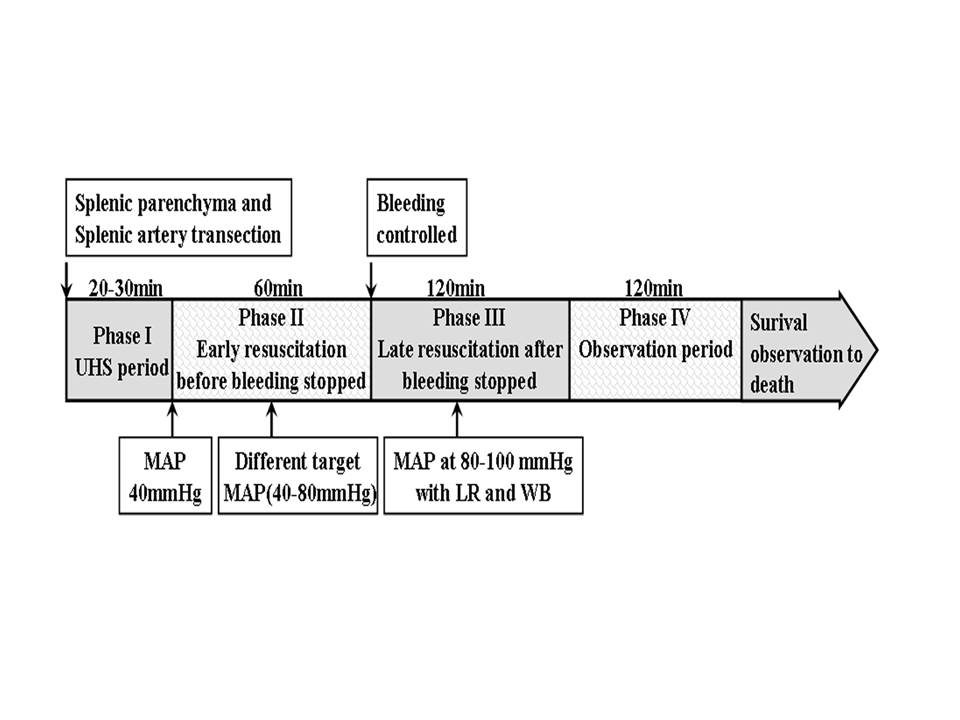
**Experiment protocol (schematic)**. LR: lactated Ringer's solution; MAP: mean artery pressure; UHS: uncontrolled hemorrhagic shock; WB: whole blood.

Phase IV was the continuous observation period (2 hours), during which the subsequent effects of different resuscitation pressure were observed. Experiments were carried out in three parts as shown below (Figure 1).

### Animal survival, fluid requirements and blood losses

Five hundred and seventy-six SD rats of different age and sex - 6 weeks (male, *n *= 96; female, *n *= 96), 14 weeks (male, *n *= 96; female, *n *= 96) or 1.5 years (male, *n *= 96; female, *n *= 96) - were divided randomly into six groups before bleeding was stopped: no treatment group, and 40-mmHg, 50-mmHg, 60-mmHg, 70-mmHg and 80-mmHg target MAP groups (*n *= 16/group). The blood loss and amount of fluid requirements to maintain the target pressure during phase II and animal survival for 24 hours were recorded. The amount of blood loss was measured at the end of phase II using the method of cotton weighing and was expressed as milliliters per kilogram. The detailed methods of animal survival observation and animal care and euthanasia are presented in Additional file 2.

### Hemodynamic parameters, tissue blood flow, organ function and blood gases

Two hundred and eighty-eight SD rats of different age and sex - 6 weeks (male,*n *= 48; female, *n *= 48), 14 weeks (male,*n *= 48; female, *n *= 48) or 1.5 years (male,*n *= 48; female, *n *= 48) - were divided randomly into six groups before bleeding was stopped: no treatment group, and 40-mmHg, 50-mmHg, 60-mmHg, 70-mmHg and 80-mmHg target MAP groups (*n *= 8/group). Animal models and fluid infusion were as described in the sections Animal Management and Experimental Phases. Hemodynamic parameters including MAP, left intraventricular systolic pressure (LVSP) and the maximal increase and decrease rate of left intraventricular systolic pressure (±dp/dt_max_), tissue blood flow in the liver, kidney and brain and their function and arterial blood gases including blood pH value, partial pressure of arterial blood oxygen (PaO_2_)and partial pressure of carbon dioxide were determined at baseline as well as at the end of phase II, phase III (definitive resuscitation) and phase IV (observation period). The detailed methods to measure these variables are presented in Additional file 3.

### Coagulationfunction

These experiments showed that different ages of hemorrhagic-shock rats had different permissive hypotension during active hemorrhage. The ideal permissive hypotension for 6-week-old, 14-week-old and 1.5-year-old rats is 40 to 50 mmHg, 50 to 60 mmHg and 70 mmHg, respectively. To understand the effects of different target resuscitation pressure on coagulation function during hypotensive resuscitation, 96 SD rats of ages 6 weeks, 14 weeks and 1.5 years (each age 32 rats, each sex 16 rats) were divided randomly into 50-mmHg and 70-mmHg target MAP groups (*n *= 8/group) to observe the effect of different target resuscitation pressure on coagulation function of hemorrhagic shock rats. The detailed procedure and measurement method are presented in Additional file 4.

### Statistical analyses

Data are presented as the mean ± standard deviation of *n *observations. Statistical differences were analyzed by three-way mixed-model analysis of variance analyses (age, sex, target resuscitation pressure), followed by the *post-hoc *Tukey test (SPSS version 15.0; SPSS Incorporated, Chicago, IL, USA) for comparison between two groups. The time and prevalence of survival were analyzed by median and interquartile ranges, Kaplan-Meier survival analyses and the log-rank test. *P *< 0.05 (two-tailed) was considered significant. Prior to analysis of variance, all data underwent the Kolmogorov-Smirnov normality test and Bartlett sphericity test; results showed that all data from different ages of rats satisfied the normality and homogeneity of variance. The sample size calculations in experiments were determined by power analysis and our previous study. The detailed analyses are presented in Additional file 5.

## Results

### Blood loss, fluid requirements and animal survival

#### Blood loss

**Figure 2:**
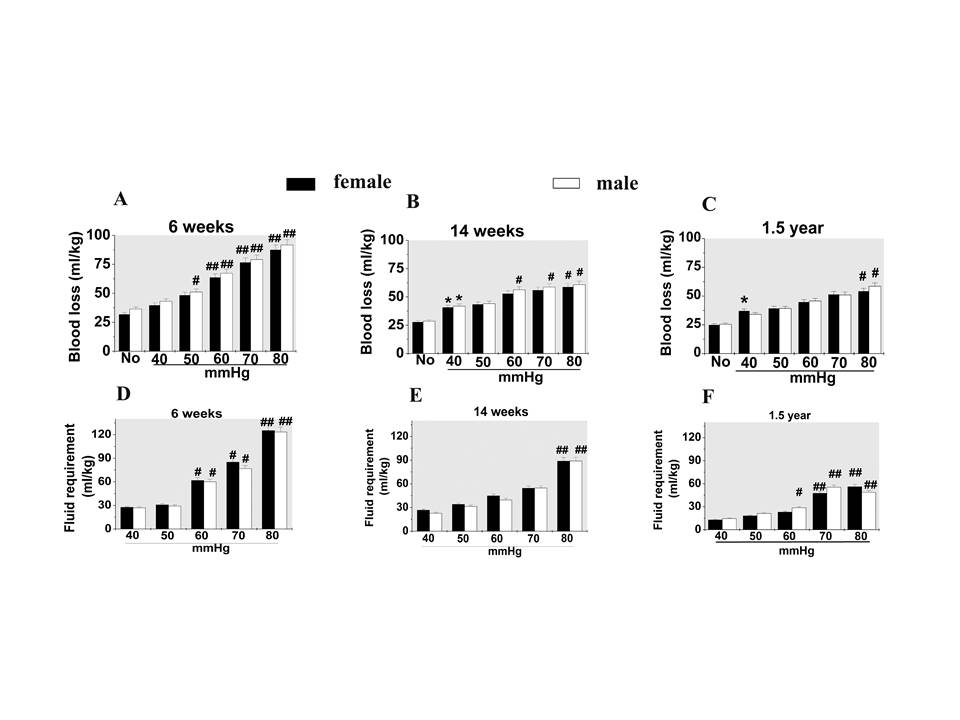
**Effects of resuscitation pressure on blood loss and fluid requirement in hemorrhagic shock**. **(A), (B), (C): **Amount of blood loss during phase I and phase II. **(D), (E), (F): **Fluid requirements to maintain the target resuscitation pressure during phase II. Data presented as mean ± standard deviation (*n *= 16/group). Analysis of variance showed there were significant differences in blood losses and fluid requirements between ages and different target resuscitation pressures (*P *< 0.01), but no significant differences between sexes (*P *> 0.05). *P < 0.05 versus no treatment group (No); #*P *< 0.05, ##*P *< 0.01 versus 40 mmHg group (*post-hoc *Tukey test).

The total blood loss during phase I and phase II had significant differences between ages and target resuscitation pressures (*P *< 0.01). Six-week-old rats had more blood loss than 14-week-old rats and 1.5-year-old rats. The 1.5-year-old rats bled least. For example, at 80 mmHg target resuscitation pressure, blood loss in 6-week-old, 14-week-old and 1.5-year-old rats was about 87.5 to 91.5 ml/kg, 58.6 to 61.5 ml/kg and 53.5 to 57.5 ml/kg, respectively. In the same age group, as the target resuscitation pressure increased, the total blood loss was significantly increased. There were no significant differences between sexes in blood loss (*P *> 0.05) (Figures 2A, B, C).

#### Fluid requirements

There were significant differences in fluid requirements between ages and target resuscitation pressures (*P *< 0.01). Six-week-old rats required more fluid than 14-week-old rats and 1.5-year-old rats. Among the three ages of rats, 1.5-year-old rats needed least fluid infusion. In the same age group, the fluid requirements were significantly increased as the target resuscitation pressure increased. There was also no significant difference in the fluid requirement between females and males in all three ages of rats (*P *> 0.05) (Figures 2D, E, F).

#### Animal survival

**Figure 3:**
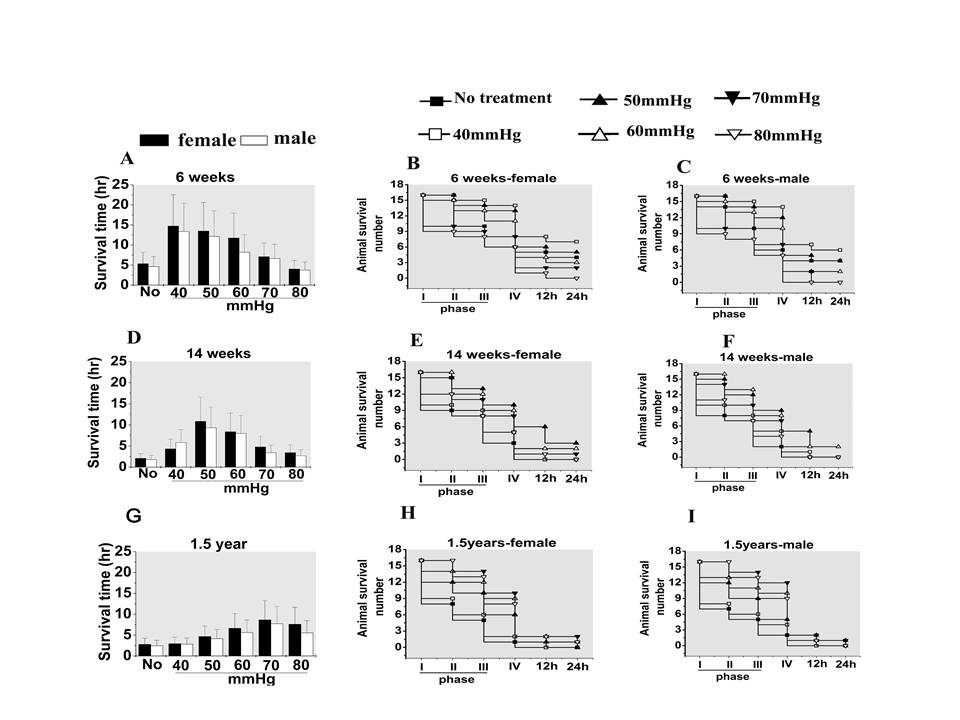
**Effects of different target resuscitation pressures on animal survival in hemorrhagic shock**. **(A), (B), (C): **Survival for 6-week-old rats. **(D), (E), (F): **Survival for 14-week-old rats.**(G), (H), (I): **Survival for 1.5-year old rats. Data presented as mean ± standard deviation (*n *= 16/group). Analysis of variance showed there were significant differences in animal survival time between ages and different target resuscitation pressures (*P *< 0.01), but no significant difference between sexes (*P *> 0.05).

There were significant differences between ages and target resuscitation pressures in survival time and 24-hour survival rate (*P *< 0.01), while there was no significant difference between sexes (*P *= 0.104)(Figure 3). For 6-week-old rats, the 40 mmHg target resuscitation pressure group had the best survival time (14.7 ± 7.8 hours and 13.9 ± 7.1 hours for male and female) and 24-hour survival rate(7/16 and 6/16 for male and female);for 14-week-old rats, the 50 mmHg target resuscitation pressure group had the best survival time(10.8 ± 5.7 hours and 9.3 ± 4.1 hours for male and female) and 24-hour survival rate(3/16 and 2/16 for male and female); and for 1.5-year-old rats, the 70 mmHg target resuscitation pressure group had the best survival time (8.6 ± 4.8 hours and 7.7 ± 4.1 hours for male and female) and 24-hour survival rate(2/16 and 1/16 for male and female). The survival time and 24-hour survival rate of 6-week-old rats was higher than for 14-week-old rats and 1.5-year-old rats (Figure 3)

### Hemodynamics, blood gases, tissue blood flow, and vital organ function

#### Hemodynamics

The baseline hemodynamic parameters of MAP, LVSP and ± dp/dt_max _had an increased trend with increasing age but no statistical significance. MAPs were 104.6 mmHg (6 weeks) and 126.2 mmHg (1.5 years), LVSPs were 131.3 mmHg (6 weeks) and 146.21 mmHg (1.5years), and +dp/dt_max _values were 5,529.8 mmHg/second (6 weeks) and 6,308.2 mmHg/second (1.5 years). These parameters were significantly decreased after hemorrhage at the end of phase I (uncontrolled hemorrhagic shock) in all age groups. During the permissive hypotension resuscitation period (phase II) and the definitive resuscitation period (phase III), all MAP values could be maintained at the set MAP level (40, 50, 60, 70 and 80 mmHg during phase II, and 80 mmHg during phase III). During the observation period (phase IV), these hemodynamic parameters demonstrated significant differences between ages and different target resuscitation pressures (*P *< 0.01), but no significant difference between sexes (*P *> 0.05). The 50-mmHg target resuscitation pressure group in 6-week-old rats and 14-week-old rats had the best MAP, LVSP and dp/dt_max _at the end of phase IV, whereas in 1.5-year-old rats the 70-mmHg target resuscitation pressure group had the best MAP, LVSP and dp/dt_max _at the end of phase IV. Since -dp/dt_max _data paralleled the +dp/dt_max _data, the data are not shown here (Tables 1, 2 and 3).

**Table 1:** Changes of mean arterial pressure (mmHg)

Group	Baseline	End of phase I	End of phase II	End of phase III	End of phase IV
**6 weeks old**					
*Female*					
No treatment	103.2 ± 10.2	39.6 ± 3.5	37.5 ± 1.8	35.6 ± 1.7	33.8 ± 1.6
40 mmHg	104.5 ± 11.2	41.2 ± 3.6	42.4 ± 2.1	83.6 ± 4.1**	82.8 ± 4.1**
50 mmHg	102.8 ± 8.5	39.5 ± 3.5	50.2 ± 2.7	85.5 ± 4.8**	90.6 ± 4.7*#
60 mmHg	104.6 ± 9.5	40.2 ± 3.5	62.8 ± 3.1	80.7 ± 4.0**	79.8 ± 3.9**
70 mmHg	106.3 ± 11.0	41.0 ± 3.6	71.5 ± 3.7	81.7 ± 3.6**	70.9 ± 3.5**
80 mmHg	108.3 ± 12.0	40.2 ± 3.5	71.4 ± 3.5	82.7 ± 3.1**	62.1 ± 3.0**
*Male*					
No treatment	104.2 ± 8.6	39.6 ± 3.5	37.5 ± 1.8	35.6 ± 1.7	33.9 ± 1.6
40 mmHg	105.2 ± 8.6	41.6 ± 3.7	42.5 ± 2.1	78.0 ± 3.8**	76.0 ± 3.7**
50 mmHg	102.3 ± 5.9	43.2 ± 3.8	51.6 ± 2.7	89.1 ± 4.4**	86.8 ± 4.3**#
60 mmHg	100.8 ± 6.8	41.5 ± 3.7	62.8 ± 3.1	85.5 ± 4.2**	79.7 ± 3.9**
70 mmHg	104.3 ± 9.4	42.2 ± 3.7	70.5 ± 3.7	76.0 ± 3.8**	70.8 ± 3.5**
80 mmHg	102.3 ± 10.2	38.9 ± 3.4	71.4 ± 3.5	76.5 ± 3.3**	62.0 ± 3.0**#
**14 weeks old**					
*Female*					
No treatment	107.8 ± 11.3	42.2 ± 3.7	40.0 ± 1.9	38.0 ± 1.8	36.1 ± 1.7
40 mmHg	108.6 ± 9.4	39.6 ± 3.5	42.4 ± 2.1	81.8 ± 3.5**	72.5 ± 3.6**
50 mmHg	106.3 ± 10.2	43.6 ± 3.9	50.8 ± 2.7	85.8 ± 4.8**	96.7 ± 4.8**##
60 mmHg	107.5 ± 9.2	41.1 ± 3.6	62.7 ± 3.1	86.8 ± 4.4**	90.7 ± 4.5**##
70 mmHg	108.9 ± 9.5	43.6 ± 3.8	69.2 ± 3.6	86.3 ± 4.3**	77.6 ± 3.9**
80 mmHg	109.8 ± 8.6	40.1 ± 3.5	81.4 ± 4.0	75.5 ± 3.7**	67.9 ± 3.3**
*Male*					
No treatment	110.2 ± 10.2	40.9 ± 3.6	38.7 ± 1.9	36.8 ± 1.8	34.9 ± 1.7
40 mmHg	109.8 ± 11.2	40.1 ± 3.5	42.4 ± 2.1	76.0 ± 3.2**	71.8 ± 3.5**
50 mmHg	110.3 ± 8.8	40.2 ± 3.5	49.5 ± 2.6	85.1 ± 4.4**	95.7 ± 4.8**##
60 mmHg	107.5 ± 8.2	41.2 ± 3.6	62.9 ± 3.1	82.6 ± 4.1**	89.7 ± 4.4**#
70 mmHg	108.6 ± 9.1	41.0 ± 3.6	72.5 ± 3.8	82.1 ± 4.1**	80.6 ± 4.0**#
80 mmHg	110.2 ± 9.6	42.0 ± 3.7	81.3 ± 4.0	81.9 ± 3.5**	70.5 ± 3.5**
**1.5 years old**					
*Female*					
No treatment	123.3 ± 11.2	39.6 ± 3.5	37.5 ± 1.8	35.7 ± 1.7	33.9 ± 1.6
40 mmHg	122.1 ± 11.5	38.5 ± 3.4	42.5 ± 2.1	84.9 ± 2.7**	53.1 ± 2.6*
50 mmHg	119.5 ± 12.6	40.3 ± 3.6	51.6 ± 2.7	83.3 ± 3.6**	70.8 ± 3.5**##
60 mmHg	118.5 ± 13.2	40.2 ± 3.5	62.8 ± 3.1	81.6 ± 3.7**	76.1 ± 3.7**##
70 mmHg	132.2 ± 8.0	40.7 ± 3.6	71.4 ± 3.7	78.4 ± 3.9**	84.9 ± 3.9**##
80 mmHg	129.6 ± 8.2	41.2 ± 3.6	81.4 ± 4.0	78.6 ± 3.4**	69.1 ± 3.4*##
*Male*					
No treatment	130.2 ± 10.2	41.0 ± 3.6	38.8 ± 1.9	36.9 ± 1.8	35.0 ± 1.7
40 mmHg	127.5 ± 7.2	39.6 ± 3.5	42.5 ± 2.1	85.7 ± 2.2**	56.3 ± 2.8*
50 mmHg	125.6 ± 6.8	40.3 ± 3.6	52.3 ± 2.8	80.9 ± 3.0**	75.1 ± 3.7**##
60 mmHg	126.9 ± 8.6	40.2 ± 3.5	62.9 ± 3.1	83.0 ± 3.6**	73.3 ± 3.6**##
70 mmHg	130.2 ± 6.2	39.6 ± 3.5	72.5 ± 3.8	81.7 ± 3.8**	79.1 ± 3.8 **##
80 mmHg	129.4 ± 9.2	39.6 ± 3.5	81.4 ± 4.05	84.3 ± 3.3**	66.5 ± 3.3**#

Data are mean ± standard deviation (*n *= 8/group). No treatment, no fluid infusion group control. Analysis of variance showed mean arterial pressure at the end of phases I and II had no significant change among all groups (among ages, genders and target resuscitation pressures), but at the end of phases III and IV there were significant differences between different target resuscitation pressures and ages (*P *< 0.01) but no significant differences between sexes. *P < 0.05; **P < 0.01versus no treatment group; #*P *< 0.05, ##*P *< 0.01 versus 40 mmHg group (*post-hoc *Tukey test).

**Table 2:** Changes of left intraventricular systolic pressure (mmHg)

Group	Baseline	End of phase I	End of phase II	End of phase III	End of phase IV
**6 weeks old**					
*Female*					
No treatment	132.9 ± 15.3	71.8 ± 8.1	68.0 ± 4.9	64.4 ± 4.6	61.1 ± 4.4
40 mmHg	133.7 ± 15.2	72.4 ± 8.2	81.9 ± 5.9	106.0 ± 7.6**	137.7 ± 9.8**
50 mmHg	134.6 ± 20.2	73.4 ± 9.6	86.7 ± 13.8	105.4 ± 9.8**	129.6 ± 11.9**
60 mmHg	130.3 ± 14.8	72.9 ± 8.3	87.6 ± 6.3	106.5 ± 7.0**	121.4 ± 8.7**
70 mmHg	128.7 ± 22.8	70.2 ± 6.8	89.5 ± 13.5	104.2 ± 18.6**	101.9 ± 15.2**
80 mmHg	130.9 ± 14.9	72.9 ± 8.3	92.0 ± 5.9	106.3 ± 6.2**	93.4 ± 6.7**#
*Male*					
No treatment	131.9 ± 15.0	66.4 ± 7.5	62.9 ± 4.5	59.6 ± 4.2	56.4 ± 4.0
40 mmHg	130.8 ± 14.8	65.8 ± 7.4	85.5 ± 6.1	107.3 ± 8.4**	130.7 ± 9.4**
50 mmHg	132.2 ± 13.6	65.4 ± 5.8	90.5 ± 11.9	108.5 ± 14.5**	123.8 ± 11.6**
60 mmHg	129.8 ± 14.7	64.2 ± 7.3	87.4 ± 6.2	106.4 ± 7.0**	116.7 ± 8.4**
70 mmHg	127.1 ± 11.6	62.9 ± 8.1	90.5 ± 9.8	104.6 ± 9.9**	100.9 ± 10.2**
80 mmHg	130.7 ± 14.8	63.6 ± 7.2	93.7 ± 5.3	107.3 ± 5.7*	92.5 ± 6.6**#
**14 weeks old**					
*Female*					
No treatment	135.6 ± 15.4	70.4 ± 8.0	66.7 ± 4.8	63.2 ± 4.5	59.9 ± 4.3
40 mmHg	133.9 ± 15.2	69.9 ± 7.9	86.0 ± 6.1	114.1 ± 8.2**	106.1 ± 7.6**
50 mmHg	128.2 ± 20.1	69.9 ± 9.0	91.1 ± 12.7	125.2 ± 17.7**	116.8 ± 16.2**
60 mmHg	133.9 ± 15.2	72.3 ± 8.2	96.1 ± 6.9	122.6 ± 8.8**	114.3 ± 8.2**
70 mmHg	134.7 ± 16.5	73.4 ± 7.6	107.4 ± 16.8	116.5 ± 16.6**	108.8 ± 16.2**
80 mmHg	132.9 ± 15.1	73.0 ± 8.3	98.3 ± 7.0	106.5 ± 7.8**	99.7 ± 7.1**
*Male*					
No treatment	132.5 ± 15.0	74.4 ± 7.3	61.0 ± 4.3	57.8 ± 4.1	54.7 ± 3.9
40 mmHg	131.2 ± 14.9	75.2 ± 7.4	96.3 ± 6.9	105.7 ± 7.5**	98.7 ± 7.11*
50 mmHg	129.9 ± 9.7	74.3 ± 8.0	101.8 ± 13.0	115.1 ± 17.0**	108.6 ± 13.3**
60 mmHg	130.9 ± 14.8	68.8 ± 7.6	98.7 ± 7.1	113.4 ± 8.1**	107.0 ± 7.7**
70 mmHg	135.3 ± 25.1	69.9 ± 4.2	99.4 ± 12.5	107.9 ± 15.3**	103.6 ± 13.9**
80 mmHg	131.4 ± 14.9	68.0 ± 7.4	98.8 ± 6.0	98.7 ± 7.1**	95.0 ± 6.8**
**1.5 years old**					
*Female*					
No treatment	143.8 ± 15.9	76.8 ± 8.7	72.8 ± 5.2	68.9 ± 4.9	65.3 ± 4.7
40 mmHg	148.7 ± 15.7	77.8 ± 8.8	98.6 ± 7.1	104.7 ± 7.4**	105.8 ± 7.6**
50 mmHg	141.9 ± 19.3	77.4 ± 3.8	104.4 ± 11.4	115.5 ± 13.2**	116.4 ± 10.1**
60 mmHg	145.7 ± 16.0	78.0 ± 8.8	104.8 ± 7.5	118.2 ± 8.5**	117.7 ± 8.4**
70 mmHg	146.1 ± 20.0	79.7 ± 7.6	106.5 ± 16.0	126.4 ± 14.9**	120.5 ± 11.7**
80 mmHg	145.7 ± 16.1	78.3 ± 8.9	107.0 ± 7.1	115.3 ± 8.2**	110.5 ± 7.6**
*Male*					
No treatment	145.4 ± 16.2	78.2 ± 8.9	74.1 ± 5.3	70.2 ± 5.0	66.5 ± 4.7
40 mmHg	148.9 ± 16.9	77.2 ± 8.7	82.6 ± 8.1	98.2 ± 7.0*	102.6 ± 7.4**
50 mmHg	146.3 ± 23.4	79.8 ± 6.5	89.3 ± 10.3	108.5 ± 11.4**	113.4 ± 11.4**
60 mmHg	145.7 ± 16.5	76.9 ± 8.7	90.9 ± 7.9	112.4 ± 8.1**	116.7 ± 8.4**
70 mmHg	148.6 ± 9.7	81.0 ± 7.8	92.5 ± 9.4	122.9 ± 15.8**	124.9 ± 11.7**
80 mmHg	147.8 ± 16.8	79.0 ± 8.9	94.8 ± 6.1	111.2 ± 8.0**	114.7 ± 8.2**

Data are mean ± standard deviation (*n *= 8/group). No treatment, no fluid infusion group control. Analysis of variance showed there were significant differences in the changes of left intraventricular systolic pressure (LVSP) between ages and different target resuscitation pressures, but no significant differences between sexes. Concretely, as age increases, the baseline LVSP is gradually increased. LVSP at the end of phases I and II had no significance between ages, sexes and pressures, but at the end of phases III and IV there were significant differences between ages and resuscitation pressures but no significant differences between sexes (*P *< 0.01).*P < 0.05; **P < 0.01 versus no treatment group; #*P *< 0.05, ##*P *< 0.01 versus 40 mmHg group (*post-hoc *Tukey test).

**Table 3:** Changes of the increase rate of left intraventricular systolic pressure (mmHg/second)

Group	Baseline	End of phase I	End of phase II	End of phase III	End of phase IV
**6 weeks old**					
*Female*					
No treatment	5,454.1 ± 717.6	2,474.2 ± 325.5	2,371.1 ± 311.9	2,272.3 ± 298.9	2,177.6 ± 286.5
40 mmHg	5,461.8 ± 718.6	2,477.2 ± 325.9	3,400.0 ± 578.9**	4,569.5 ± 601.2**	5,045.9 ± 663.9**
50 mmHg	5,905.4 ± 1,362.2	2,314.5 ± 1,114.4	4,663.8 ± 918.0**#	4,777.2 ± 903.7**	5,275.2 ± 601.9**
60 mmHg	5,476.4 ± 720.5	2,482.9 ± 326.7	4,602.1 ± 605.5**#	4,453.3 ± 585.9**	4,878.6 ± 641.9**
70 mmHg	5,410.0 ± 788.7	2,456.9 ± 645.3	4,776.7 ± 1,111.0**#	4,420.9 ± 1,015.7**	4,821.9 ± 932.0**
80 mmHg	5,547.3 ± 729.9	2,510.8 ± 330.3	4,525.3 ± 595.4**#	4,188.2 ± 551.0**	4,568.2 ± 601.0**#
*Male*					
No treatment	5,417.0 ± 712.7	2,118.5 ± 278.7	2,030.2 ± 267.1	1,945.7 ± 256.0	1,864.6 ± 245.3
40 mmHg	5,424.8 ± 713.7	2,121.2 ± 279.1	3,519.6 ± 528.9**	4,404.9 ± 579.6**	4,943.3 ± 650.4**
50 mmHg	5,938.1 ± 833.1	2,013.5 ± 681.6	4,278.2 ± 538.1**	4,605.2 ± 398.8**	5,168.0 ± 459.9**
60 mmHg	5,439.4 ± 715.7	2,126.1 ± 279.7	4,251.1 ± 559.3**	4,525.9 ± 595.5**	4,842.5 ± 637.1**
70 mmHg	5,372.7 ± 638.0	2,103.5 ± 522.0	4,328.0 ± 386.0**	4,518.0 ± 499.0**	4,796.0 ± 558.0**
80 mmHg	5,510.8 ± 725.1	2,150.3 ± 282.9	4,100.2 ± 539.5**	4,280.2 ± 563.1**	4,543.5 ± 597.8**
**14 weeks old**					
*Female*					
No treatment	5,925.4 ± 779.6	2,750.0 ± 361.8	2,635.4 ± 346.7	2,525.6 ± 332.3	2,420.3 ± 318.4
40 mmHg	5,933.7 ± 780.7	2,753.5 ± 362.3	3,491.2 ± 590.9**	5,068.1 ± 666.8**	4,898.9 ± 644.6**
50 mmHg	6,369.5 ± 1,402.5	2,654.6 ± 1,147.7	4,232.5 ± 731.3**	5,298.4 ± 930.4**	5,121.6 ± 268.0**
60 mmHg	5,949.4 ± 782.8	2,760.0 ± 363.1	4,852.7 ± 638.5**	4,943.2 ± 650.4**	4,932.7 ± 649.0**
70 mmHg	5,877.8 ± 927.1	2,730.2 ± 758.5	4,392.3 ± 1,007.3**	4,907.7 ± 1,578.3**	4,905.7 ± 1,407.8**
80 mmHg	6,026.0 ± 792.8	2,791.9 ± 367.3	4,161.1 ± 547.5**	4,649.4 ± 611.7**	4,647.5 ± 611.5**
*Male*					
No treatment	6,689.7 ± 880.2	2,321.2 ± 305.4	2,224.5 ± 292.7	2,131.8 ± 280.5	2,042.9 ± 268.8
40 mmHg	6,698.2 ± 881.3	2,324.1 ± 305.8	3,379.9 ± 576.3**	4,696.6 ± 617.9**	4,592.4 ± 604.2**
50 mmHg	6,495.3 ± 1,417.3	2,231.5 ± 660.0	4,660.1 ± 413.9**#	4,910.0 ± 910.3**	4,801.1 ± 645.3**
60 mmHg	6,714.2 ± 883.4	2,329.6 ± 306.5	4,814.1 ± 633.4**#	4,674.9 ± 615.1**	4,693.2 ± 617.5**
70 mmHg	6,641.3 ± 849.6	2,304.5 ± 695.1	4,224.5 ± 944.6**	4,651.4 ± 823.9**	4,677.8 ± 563.1**
80 mmHg	6,792.3 ± 893.7	2,356.4 ± 310.0	4,002.1 ± 526.6**	4,406.6 ± 579.8**	4,431.6 ± 583.1**
**1.5 years old**					
*Female*					
No treatment	6,664.3 ± 876.8	2,777.2 ± 365.4	2,661.5 ± 350.2	2,550.6 ± 335.6	2,444.3 ± 321.6
40 mmHg	6,672.2 ± 877.9	2,780.9 ± 365.9	3,398.3 ± 447.1**#	4,711.4 ± 619.9**	5,625.3 ± 740.1**
50 mmHg	6,002.8 ± 1,707.0	2,803.2 ± 1,397.9	4,230.9 ± 594.2**##	5,104.0 ± 1,121.5**	6,094.0 ± 931.6**
60 mmHg	6,687.0 ± 879.8	2,787.8 ± 366.8	4,492.9 ± 591.1**##	5,208.6 ± 685.3**	6,307.0 ± 829.8**
70 mmHg	6,619.5 ± 1,166.2	2,756.3 ± 954.1	4,710.4 ± 827.6**##	5,295.4 ± 1,312.6**	6,483.7 ± 1,638.6**
80 mmHg	6,759.1 ± 889.3	2,821.5 ± 371.2	4,462.5 ± 587.1**##	5,016.7 ± 660.1**	6,142.5 ± 808.2**
*Male*					
No treatment	5,961.5 ± 784.4	2,618.5 ± 344.5	2,509.4 ± 330.2	2,404.9 ± 316.4**	2,304.7 ± 303.2**
40 mmHg	5,969.8 ± 785.5	2,622.0 ± 345.0	3,418.3 ± 449.7**	4,787.5 ± 629.9**	5,711.2 ± 751.4**
50 mmHg	6,400.8 ± 1,410.1	2,654.6 ± 1,154.0	4,445.3 ± 761.2**	5,186.4 ± 1,559.6**	6,187.1 ± 1,311.6**
60 mmHg	5,985.6 ± 787.5	2,628.6 ± 345.8	4,719.6 ± 621.0**#	5,197.9 ± 683.9**	6,296.6 ± 828.5**
70 mmHg	5,913.7 ± 1,381.5	2,598.7 ± 1,130.8	4,947.3 ± 1,040.2**#	5,207.4 ± 781.8**	6,387.5 ± 1,125.5**
80 mmHg	6,062.6 ± 797.7	2,660.5 ± 350.0	4,686.9 ± 616.7**#	4,933.3 ± 649.1**	6,051.3 ± 796.2**

Data are mean ± standard deviation (*n *= 8/group). No treatment, no fluid infusion group control. Analysis of variance showed significant differences in the changes of the increase rate of left intraventricular systolic pressureafter hemorrhagic shock and fluid infusion between ages and different target resuscitation pressures (*P *< 0.01), but no significant difference between sexes (*P *> 0.05). **P < 0.01 versus no treatment group; #*P *< 0.05, ##*P *< 0.01 versus 40 mmHg group (*post-hoc *Tukey test).

#### Blood gases

The normal values of blood pH, PaO_2 _and partial pressure of carbon dioxide showed no differences in the different age and sex groups. The blood pH in all groups of rats showed the same extent of decrease during phase II as compared with phase I. During phases III and IV, blood pH had some extent of recovery in all resuscitation pressure groups in all ages of rats. Blood pH in rats at 6 weeks and 14 weeks of age in the 50-mmHg target resuscitation pressure group as well as in 1.5-year-old rats in the 70-mmHg target resuscitation pressure group recovered better than in other target resuscitation pressure groups. The partial pressure of carbon dioxide in all groups had the same extent of decrease after hemorrhagic shock, but showed no significant change after fluid infusion and there was no significant difference in all groups. The PaO_2 _of all rats was slightly reduced after hemorrhage (at the end of phase I), and was significantly increased during phases II, III and IV. Also, PaO_2 _in6-week-old and 14-week-old rats in the 50-mmHg target resuscitation pressure group and in 1.5-year-old rats in the 70-mmHg target resuscitation pressure group was higher than in other target resuscitation pressure groups of rats of the same age.

The overall analyses showed there were significant changes in the changes of blood gases following hemorrhagic shock and fluid infusion between target resuscitation pressure groups (*P *< 0.01), but no significant difference between ages and sexes (*P *> 0.05). The 50-mmHg target resuscitation pressure group of 6-week-old and 14-week-old rats and the 70-mmHg target resuscitation pressure group of 1.5-year-old rats had the best blood gas parameters (Additional file 6).

#### Blood flow in vital organs

**Figure 4:**
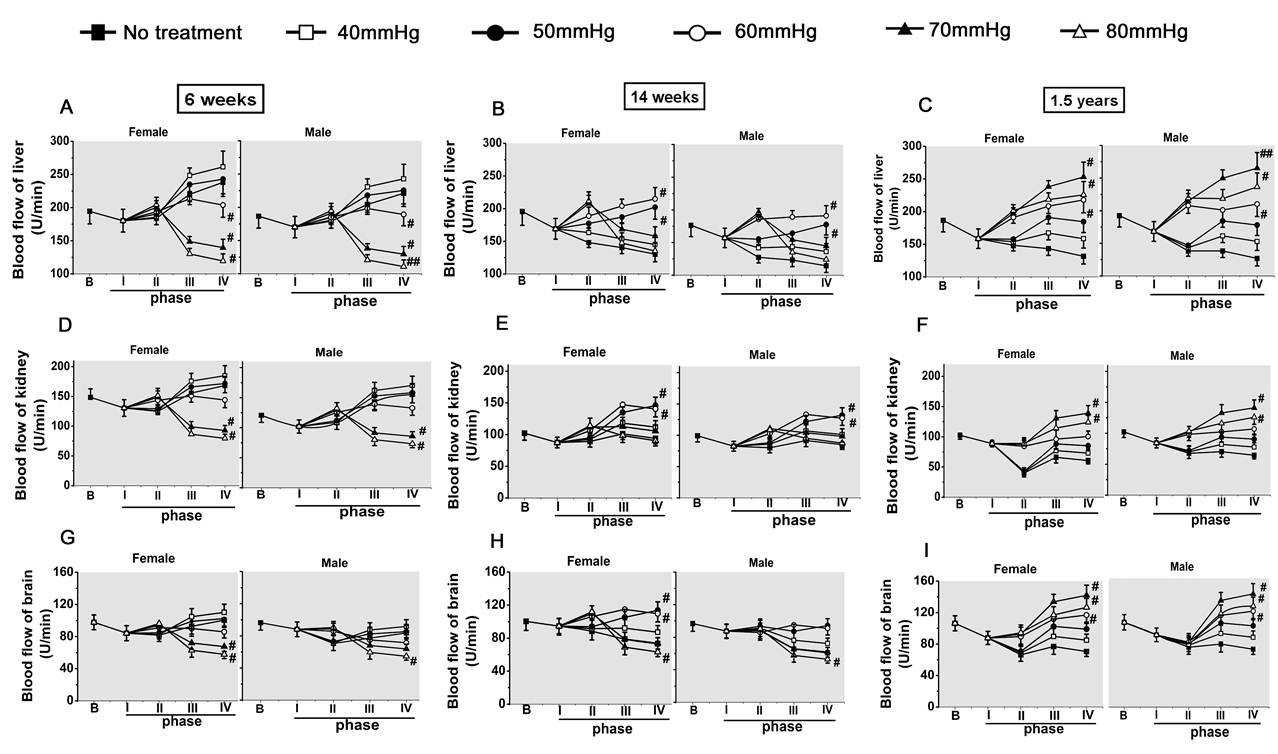
**Effects of different target resuscitation pressures on tissue blood flow in the liver, kidney and brain**. **(A), (B), (C): **Liver blood flow in 6-week-old rats, 14-week-oldrats and 1.5-year-old rats. **(D), (E), (F): **Kidney blood flow in 6-week-old rats, 14-week-oldrats and 1.5-year-old rats. **(G), (H), (I): **Brain blood flow in 6-week-old rats, 14-week-oldrats and 1.5-year-old rats. Data presented as mean ± standard deviation (*n *= 8/group). Analysis of variance showed there were significant differences in the changes of tissue blood flow of the liver, kidney and brain after fluid infusion between ages and different target resuscitation pressures (*P *< 0.01), but no significant difference between sexes (*P *> 0.05). #*P *< 0.05, ##*P *< 0.01 versus 40 mmHg group (*post-hoc *Tukey test).

There were significant differences in the changes of blood flow of the liver, kidney and brain following hemorrhagic shock and fluid infusion between ages and target resuscitation pressures (*P *< 0.01), while there was no significant difference between sexes (*P *> 0.05). Concretely, the blood flow in the liver, kidney and brain was significantly decreased at the end of phase I (after hemorrhage) (Figure 4). The average reduction rates of the liver, kidney and brain were 10.8% for 6-week-old rats, 11.9% for 14-week-old rats and 15.8% for 1.5-year-old rats, respectively. The reduction in the liver and kidney was more obvious. At the end of phase II, the blood flows in the liver, kidney and brain were increased with the increase of target resuscitation pressure in three ages of rats. At the end of phase III and phase IV, the 40-mmHg to 50-mmHg target resuscitation group in 6-week-old rats, the 50-mmHg to 60-mmHg target resuscitation group in 14-week-old rats and the 70-mmHg target resuscitation group in 1.5-year-old rats had higher blood flow in the liver, kidney and brain. There were no significant differences between sexes in all ages of rats (*P *> 0.05) (Figure 4).

#### Liver and kidney function

The levels of alanine aminotransferase, aspartate aminotransferase, and blood urea nitrogen in blood as well as serum creatinine had some extent of increase after hemorrhage. At the end phase II and phase IV, the variables of liver and kidney function were increased further. The 40-mmHg to 50-mmHg target pressure group in 6-week-old rats, the 50-mmHg to 60-mmHg target pressure group in 14-week-old rats, and the 60-mmHg to 70-mmHg target pressure group in 1.5-year-old rats had lower concentrations of alanine aminotransferase, aspartate aminotransferase, blood urea nitrogen and serum creatinine than the other target pressure groups at the same age. Overall, there were significant differences in the changes of liver and renal function following hemorrhagic shock and fluid infusion between ages and target resuscitation pressures following hemorrhagic shock and fluid infusion (*P *< 0.01), while there was no significant difference between genders (Additional file 7).

### Coagulation function

Compared with baseline, the thrombin time(TT), prothrombin time (PT) and activated partial thromboplastin time (APTT) were slightly prolonged, the International Normalized Ratio of prothrombin time(PT-INR) slightly deceased, and fibrinogen concentration did not change obviously at the end of phase I in all ages of rats. At the end of phases II, III and IV, the TT, PT and APTT were further prolonged, PT-INR significantly increased, and fibrinogen concentration slightly increased. All of these coagulation parameters showed no differences in different target resuscitation pressure groups in the same age groups of rats and demonstrated no differences in different ages and sexes of rats (*P *> 0.05) (Additional file 8).

## Discussion

Aggressive fluid resuscitation for uncontrolled hemorrhagic shock before bleeding can cause significant blood loss, severe hemodilution, clot dislocation and loss of platelets and coagulant factors, thereby increasing the risk of death. Limited/hypotensive resuscitation has been considered to be a better resuscitation approach for such uncontrolled hemorrhagic shock. Studies have demonstrated that a target blood pressure of 50 to 60 mmHg before control of bleeding after uncontrolled hemorrhagic shock is an ideal resuscitation pressure for adult rats [7-9]. However, the ideal resuscitation pressure for pre-adults and older individuals after uncontrolled hemorrhagic shock is not known.

Using uncontrolled hemorrhagic-shock rats of different ages and sexes (6 weeks, 14 weeks and 1.5 years representing pre-adult, adult and older rats, respectively), we compared the resuscitation effects of different target pressures (40, 50, 60, 70 and 80 mmHg) on uncontrolled hemorrhagic shock during active hemorrhage and the age and sex differences. The results showed that different target resuscitation pressure had different resuscitation outcome for the same age and sex of rats, while for all ages and sexes of rats the resuscitation effects had significant differences between ages and target resuscitation pressures but no significant difference between sexes. Each age of rats had a relative appropriate different target pressures (optimal different target pressures):40 to 50 mmHg for 6-week-old rats, 50 to 60 mmHg for 14-week-old rats and 70 mmHg for1.5-year-old rats. Fluid infusion with this target pressure obtained a good resuscitation effect in improving animal survival, hemodynamics, organ function and so forth, as compared with other target resuscitation pressures.

The present study further confirmed that 50 to 60 mmHg is the ideal target resuscitation pressure for uncontrolled hemorrhagic shock in adult individuals [7,8]. The study also showed that pre-adults and older individuals who had suffered trauma had their own ideal target resuscitation pressure during the active hemorrhage. The ideal target resuscitation pressure of pre-adults (40 to 50 mmHg) was lower than in adults (50 to 60 mmHg) and older individuals (70 mmHg). There may be multiple reasons for these findings. The first reason is that adult and younger individuals have stronger cardiovascular function and adaptive abilities than older individuals. For example, studies have shown that maximal cardiac output, peripheral vascular reserve and vascular conductance decline in older women and men as compared with younger individuals [19-23]. The second reason is that adult and younger individuals have stronger tolerance to ischemia and hypoxia. Hagberg and colleagues found that, as compared with older persons, young men had stronger tolerance to hypoxia induced by exercise [21]. The third reason is that older individuals have reduced immune function, which can damage the ability of host to fight against insults. Heinrich and colleagues found improved immune function can lead to less shock-induced tissue damage [15,24,25]. This result also suggests that, for older patients who have suffered trauma, apart from increasing the target resuscitation pressure before active bleeding has been stopped, cardiovascular (and other organ) function should be focused upon.

The present study also found that different ages of rats needed different amounts of fluid infusion to maintain their target resuscitation pressure; the younger rats needed more fluid than older rats. The reasons for this may include: the younger rats lost more blood than the older rats during the period of phase I, so they needed more fluid infusion to maintain the same level of target resuscitation pressure than older rats; or, the compliance of blood vessels in younger rats is better than that in the older rats, and good compliance of blood vessels can accommodate more fluid [26]. Another phenomenon observed in the present study was that the ideal target resuscitation pressure for each age of rats was not too high. The reason for this may be that maintaining higher target pressure needed more fluid infusion; a large amount of fluid infusion can cause fluid overload and more blood loss and hemodilution. These factors may aggravate tissue ischemia and damage, including the reduction of cardiac contractility.

Traumatic coagulopathy is a hypocoagulable state that often occurs in the most severely injured. There are multiple factors that may contribute to coagulopathy after severe trauma or shock. These include the increase of anticoagulation factors such as hyperfibrinolysis and tissue plasminogen activator, and the decrease in the concentration of plasminogen activator and thrombin activatable fibrinolysis inhibitor [27,29]. In addition, hemodilution induced by excessive infusion of crystalloids and banked red blood cells can worsen shock-induced hypocoagulation [30-32]. Our previous study and other studies showed that permissive hypotensive resuscitation had no obvious influences on coagulation [7]. The present study found that coagulation parameters including the PT, APTT, PT-INR and TT had a significant hemodilution-induced changes, but not for fibrinogen concentration. The reason for this may be because the PT, APTT, PT-INR and TT are all coagulation time-related variables; many factors including the changes of coagulation factors and cytokines and the acid-base balance disorder after hemorrhage and hemodilution can affect them, so their changes were great after hemorrhage and fluid resuscitation [28-30]. While fibrinogen is a single factor, the effect is not great just from hemodilution. The compensatory reaction induced by short duration of hemorrhage [33] may neutralize hemodilution-induced decrease of fibrinogen, so the concentration of fibrinogen changed was not great after fluid infusion. Of course, the precise reason needs further investigation.

Although we found that different ages of rats had their own ideal target pressure to resuscitate uncontrolled hemorrhagic shock before bleeding was controlled, this finding provided a strong basis for the personalization of the fluid strategy for different ages of trauma patients. The present study had several limitations. First, this study was limited to small and anesthetized animals; whether this model can completely reflect uncontrolled hemorrhagic shock in humans and whether this ideal target MAP in animals is suitable to for humans need confirmation. Second, the solution used was lactated Ringer's solution +6% hydroxyethyl starch-130; whether other solutions, such as hypertonic solution, are more suitable for different ages requires further study. Third, the relationship of large amount of fluid infusion to cardiac contractility and the precise effect of hypotensive resuscitation on coagulation function following hemorrhagic shock need further investigation.

## Conclusion

Different ages of rats have different permissive hypotension to resuscitate uncontrolled hemorrhagic shock during the active hemorrhage. The ideal target resuscitation pressure for uncontrolled hemorrhagic shock in pre-adult rats (6 weeks old), adult rats (14 weeks old) and older rats (1.5 years old) was 40 to 50 mmHg, 50 to 60 mmHg and 70 mmHg, respectively. Their resuscitation effects have significant age difference but had no significant sex difference.

## Key messages

• The target resuscitation pressure for uncontrolled hemorrhagic shock is different in different ages of rats.

• Older rats need a higher target resuscitation pressure (70 mmHg) than reproductive-age rats (50 to 60 mmHg) during uncontrolled hemorrhagic shock.

## Abbreviations

APTT: activated partial thromboplastin time; ±dp/dt_max_: maximal increase and decrease rate of left intraventricular pressure; LVSP: left ventricular systolic pressure; MAP: mean arterial pressure; PaO_2_: partial pressure of arterial blood oxygen; PT: prothrombin time; PT-INR: International Normalized Ratio of prothrombin time; SD: Sprague-Dawley; TT: thrombin time.

## Competing interests

The authors declare that they have no competing interests.

## Authors' contributions

LT participated in the design of the study, the statistical analysis, the entire experiment, and the preparation of the manuscript. ZY, TKL, XMY, PXY and LD participated in the entire experiment and data collection and analysis. LLM conceived of the study, and participated in the design and coordination and edited the manuscript. All authors read and approved the final manuscript.

## Supplementary Material

Additional file 1 Additional file 1 **is a document presenting further animal management details**. Click here for file

Additional file 2 Additional file 2 **is a document presenting further animal survival, fluid requirement and blood loss details**. Click here for file

Additional file 3 Additional file 3 **is a document presenting further hemodynamic parameter, tissue blood flow, organ function and blood gas details**. Click here for file

Additional file 4 Additional file 4 **is a document presenting further coagulation function details**. Click here for file

Additional file 5 Additional file 5 **is a document presenting further statistical analysis details**. Click here for file

Additional file 6 Additional file 6 **is Table S1 presenting the changes of pH, Table S2 presenting the changes of partial pressure of carbon dioxide (mmHg), and Table S3 presenting the changes of PO_2 _(mmHg)**. Click here for file

Additional file 7 Additional file 7 **is Figure S1 showing effects of different target resuscitation pressures on liver and renal function in different ages and sexes of rats after uncontrolled hemorrhagic shock**. Data are mean ± standard deviation (*n*= 8/group). (A) changes in alanine aminotransferase (ALT) levels; (B) changes in aspartate aminotransferase (AST) level; (C) changes in blood urea nitrogen (BUN); (D) changes in serum creatinine (Scr). Analysis of variance showed these parameters had significant changes following hemorrhagic shock and fluid infusion between ages and different target resuscitation pressures(*P *< 0.01), but no significant difference between sexes (*P *> 0.05). #*P *< 0.05, ##*P *< 0.01 versus 40 mmHg group. Click here for file

Additional file 8 Additional file 8 **is Table S4 presenting the effects of different pressures on coagulation parameters**. Click here for file
