# Supplementary material for: Ideal resuscitation pressure for uncontrolled hemorrhagic shock in different ages and sexes of rats
Source: Crit Care. 2013 Sep 10;17(5):R194. doi: 10.1186/cc12888 (PMC4264615; doi:10.1186/cc12888)
Supplement: Additional file 1 — is a document presenting further animal management details. [file cc12888-S1.docx]

# Animal management

Sprague–Dawley (SD) rats of 6 weeks (body weight, female: 114±9.2g; male: 115±19.9), 14 weeks (body weight,female: 209±9.3; male:230±14.9) or 1.5 years (body weight, female: 388±39.7; male: 457 ±59.2) were fasted for 12 h but allowed water with sugar-salt solution (5% glucose saline) *ad libitum* before the experiment. On the day of experiment, rats were first anesthetized with sodium pentobarbital (30 mg/kg, ip). This was added until the rats had no response to a needle stimulus of front toe, the total amount of sodium pentobarbital was ~50 mg/kg. None of rats appeared apnea under this anesthesia. The right femoral artery and left and right femoral vein were catheterized with a polyethylene catheter (outer diameter, 0.96 mm; inner diameter 0.58 mm) for monitoring the mean arterial blood pressure (MAP) with Polygraph Physiological Recorder (SP844, Power Laboratory; AD Instruments, Castle Hill, NSW, Australia), bleeding and infusion. Left ventricular catheterization *via* the right carotid artery was done for observation of hemodynamics. To prevent clot formation, the artery catheter was filled with normal (0.9%) saline containing 30 U/mL of heparin. To maintain the body temperature at 37°C (monitored by anus thermometer), rats were placed on a warming plate. An uncontrolled hemorrhagic-shock model was reproduced as described before in our research team.[7,8]. Breifly, after the completion of catheterization, the abdomen was disinfected and a laparotomy was performed. The spleen was exposed and a cross-transection was made in the splenic parenchyma between the two major branches of the splenic artery (the transected spleen was removed but not leave in abdomen). Meanwhile, one of the major branches of the splenic artery and vein were also transected. Blood was allowed to flow into the abdominal cavity. When the MAP decreased to 40 mmHg (20-30 min), uncontrolled hemorrhagic shock was established for subsequent experiments. After the model production, the abdomen was not completely closed, but simply closed with suture and dressed with wet gauze. For animal survival and coagulation observation, after splenic artery and vein were ligated at the end of phase II, the abdomen was completely closed with suture, while for hemodynamics and tissue blood flow observation, the abdomen was not completely closed till the end of phase IV, before which, the abdomen just simply closed and dressed with wet gauze for observation of blood flow of liver and kidney.

The rats used in present study were supplied by Animal Center of Daping Hospital, Third Military Medical University. It is Sprague Dawley rat. The licence number for the manufacture and supplier are SCXK(YU)2012-005 and SYXK(YU)-2012-0010. Rats are housed in SPF animal house. The cage type is 80×50×60cm, each cage have eight rats. The husbandry temperature is 20-25℃, humidity is 40-70%. The intensity of illumination is 15-20 and in turn for 12 hours. The food for rats include (%): corn 35%, soybean 16%, wheat 15%, wheat meal 17.74%, yeast 2%, bone meal 2.5%, sesame 7%, fish meal 1%, milk powder 2.0%, vegetable oil 1%, sale 0.5%, multivitamins 0.06%, microelement 0.2%. The detailed materials and methods have been put into the supplemental data.
