# Supplementary material for: Ideal resuscitation pressure for uncontrolled hemorrhagic shock in different ages and sexes of rats
Source: Crit Care. 2013 Sep 10;17(5):R194. doi: 10.1186/cc12888 (PMC4264615; doi:10.1186/cc12888)
Supplement: Additional file 2 — is a document presenting further animal survival, fluid requirement and blood loss details. [file cc12888-S2.docx]

**Animal survival, fluid requirements and blood losses**

Five hundred seventy six SD rats of different age and sex: 6 weeks (male:96, female 96) , 14 weeks (male:96, female 96) or 1.5 year (male:96, female 96) were divided randomly into six groups before bleeding was stopped: no-treatment group, 40-, 50-, 60-, 70-, 80-mmHg target MAP groups (n=16/group). The blood loss and amount of fluid requirements to maintain the target pressure during phase II and animal survival for 24 hours were recorded. The amount of blood loss was measured at the end of phase II using the method of cotton weighing and was expressed as ml/kg. The animal survival was monitored by artificial observation after removing the cannula and closing the abdomen. The rats were put back to their cage and allowed to recover consciousness during the period of observation. The zero time of survival observation was the end of phase I. To relieve the pain of post-operation, Jingsongling (xylidinothiazole, 0.15 mg/kg) was injected intramuscularly. Rats were in articulo mortis during the period of survival observation or survived over 24 hours, they would be euthanized by giving over dose of sodium pentobarbital.
