# Supplementary material for: Ideal resuscitation pressure for uncontrolled hemorrhagic shock in different ages and sexes of rats
Source: Crit Care. 2013 Sep 10;17(5):R194. doi: 10.1186/cc12888 (PMC4264615; doi:10.1186/cc12888)
Supplement: Additional file 3 — is a document presenting further hemodynamic parameter, tissue blood flow, organ function and blood gas details. [file cc12888-S3.docx]

**Hemodynamic parameters, tissue blood flow, organ function and blood gases**

Two hundred and eighty eight SD rats of different age and sex: 6 weeks (male 48, female 48), 14 weeks (male 48, female 48) or 1.5 year (male 48, female 48) were divided randomly into six groups before bleeding was stopped: no-treatment group, and 40-, 50-, 60-, 70-, 80-mmHg target MAP groups (n=8/group). Animal models and fluid infusion were as described in the section of animal management and experimental phases. Hemodynamic parameters including MAP, left intra-ventricular systolic pressure(LVSP) and the maximal increase and decrease rate of left intra-ventricular systolic pressure(±dp/dtmax), tissue blood flow in the liver, kidney and brain and their function and arterial blood gases including blood pH value, partial pressure of arterial oxygen (PaO_2)_ and carbon dioxide (PCO_2_) were determined at baseline as well as at the end of phase II, phase III (definitive resuscitation) and phase IV (observation period). The hemodynamics was monitored by a Polygraph Physiological Recorder (SP844, Power Laboratory; AD Instruments, Castle Hill, NSW, Australia) via the left ventricular catheter of heart. The blood flows of liver, kidney and brain were measured by a Laser Doppler Blood Flowmeter (Periflux system 5000, Primed, Stockholm, Sweden) via flowmeter probe (probe 403, ID: 021205). The probes were put on the center of the middle leaf of liver and parenchyma of left kidney (as the representative of kidney) to measure the liver and kidney blood flow. For the measurement of brain blood flow, a small hole was made on parietal bone by the side of sagittal suture. The variables of liver and kidney function were measured by a Biochemical Analyzer (DX800, Biochemical Analyzer, Beckman, Fullerton, CA, USA). The blood gases were measured by a Blood Gas Analyzer (Phox plus L; Nova Biomedical, Waltham, MA, USA). In order to measure the liver and kidney blood flow, during the period of experiment, the abdomen was only simply closed and covered with wet gauze. The volumes of the blood samples for the blood gases and organ function tests were 0.3ml and 0.5 ml respectively. To avoid the additional blood loss for rats, equal volumes of blood were supplied after each sample taken.
