# Supplementary material for: Ideal resuscitation pressure for uncontrolled hemorrhagic shock in different ages and sexes of rats
Source: Crit Care. 2013 Sep 10;17(5):R194. doi: 10.1186/cc12888 (PMC4264615; doi:10.1186/cc12888)
Supplement: Additional file 4 — is a document presenting further coagulation function details. [file cc12888-S4.docx]

**Coagulation function**

Above experiments showed that different ages of hemorrhagic-shock rats had different permissive hypotension during active hemorrhage. The “ideal” permissive hypotension for 6-week, 14-week and 1.5-year-old rats is 40–50 mmHg, 50–60 mmHg and 70 mmHg, respectively. To understand the effects of different target resuscitation pressure on coagulation function during hypotensive resuscitation, 96 SD rats of ages 6 weeks, 14 weeks and 1.5 year(each age 32 rats, each sex 16 rats) were divided randomly into 50-, and 70-mmHg target MAP groups (n=8/group). The procedures of shock model production and fluid resuscitation were the same as above. Blood was sampled (200 ul/ each time, citrate: blood 1:9) at baseline as well as at the end of phase I, II, III and 2 h of phase IV to measure coagulation parameters including the thrombin time (TT), prothrombin time (PT), international normalized ratio of prothrombin time (PT-INR), activated partial prothrombin time (APTT), and fibrinogen by coagulation function Analyzer (Acl-top 700, Beckman, Fullerton, CA, USA) To avoid the additional blood loss for the rats, equal volumes of blood were supplied after each sample taken. To avoid the interference of heparin on coagulation function, heparin was not used in this part of the experiment.
