# Supplementary material for: Ideal resuscitation pressure for uncontrolled hemorrhagic shock in different ages and sexes of rats
Source: Crit Care. 2013 Sep 10;17(5):R194. doi: 10.1186/cc12888 (PMC4264615; doi:10.1186/cc12888)
Supplement: Additional file 5 — is a document presenting further statistical analysis details. [file cc12888-S5.docx]

**Statistical analyses**

Data were presented as the mean ± SD of n observations. Statistical differences were analyzed by three-way mixed-model ANOVA analyses (age, sex, target resuscitation pressure), followed by the *post-hoc* Tukey test (SPSS ver15.0, SPSS Incorporated, Chicago, IL, USA) for some comparison between two groups. The time and prevalence of survival were analyzed by median and interquartile ranges (IQRs), Kaplan–Meier survival analyses and the log rank test. *P*<0.05 (two-tailed) was considered significant. The sample size calculation were mainly based our previous study and expected treatment effects by power analysis (power set at 80%, alpha at 0.05, beta at 0.20). The method we used was ANOVA. Since the present study involved many parameters including animal survival, hemodynamic parameters (MAP, LVSP, +/-dp/dttmax), and coagulation function. The expected effect and difference for each parameter is different, so the sample size calculation was different for different experiment. Based on our previous study, the expected differences for survival rate and survival time among different target resuscitation pressure were about 40%-60%, the expected differences for hemodynamics parameters (such as MAP, LVSP, +/-dp/dt max) among different target resuscitation pressure were about 20%-40%, taken together with our pilot study and power calculation, 16 rats/group were adopted in the present study to observe the animal survival, and 8 rats/group were used to observe the hemdynamics and other parameters. In addition, for sample size calculation we did not count for sex, just considered the total animal number in each group. The ideal resuscitation pressures for each age of rats were determined by assessing the animal survival, hemodynamics and organ function among all target resuscitation pressures. Prior to ANOVA analysis, all data received Kolmogorov-Smirnov normality test and Bartlett sphericity test, results showed all data from different ages of rats satisfied the normality and hemogeneity-of-variance.
