# Supplementary material for: Ideal resuscitation pressure for uncontrolled hemorrhagic shock in different ages and sexes of rats
Source: Crit Care. 2013 Sep 10;17(5):R194. doi: 10.1186/cc12888 (PMC4264615; doi:10.1186/cc12888)
Supplement: Additional file 6 — is Table S1 presenting the changes of pH, Table S2 presenting the changes of partial pressure of carbon dioxide (mmHg), and Table S3 presenting the changes of PO2 (mmHg). [file cc12888-S6.docx]

**Table S 1.** **The changes of pH**

| **group** | | **baseline** | **End of phase I** | **end of phase II** | **end of phase III** | **end of phase IV** |
| --- | --- | --- | --- | --- | --- | --- |
| **6weeks**  **-female** | **No Treat** | 7.373±0.043 | 7.379±0.036 | 7.144±0.046+ | 7.196±0.046 | 7.242±0.046 |
|  | **40mmHg** | 7.335±0.046 | 7.341±0.046 | 7.192±0.054+ | 7.244±0.055 | 7.290±0.055 |
|  | **50mmHg** | 7.411±0.061 | 7.416±0.061 | 7.229±0.073+ | 7.281±0.061 | 7.328±0.050 |
|  | **60mmHg** | 7.373±0.055 | 7.378±0.055 | 7.246±0.059 | 7.287±0.060 | 7.323±0.060 |
|  | **70mmHg** | 7.334±0.061 | 7.340±0.061 | 7.288±0.021 | 7.301±0.063 | 7.312±0.099 |
|  | **80mmHg** | 7.409±0.052 | 7.411±0.052 | 7.325±0.060 | 7.298±0.060 | 7.275±0.060 |
| **6weeks**  **-male** | **No Treat** | 7.342±0.044 | 7.330±0.065 | 7.188±0.046+ | 7.193±0.046+ | 7.197±0.046 |
|  | **40mmHg** | 7.303±0.045 | 7.292±0.045 | 7.236±0.055 | 7.241±0.055 | 7.245±0.055 |
|  | **50mmHg** | 7.330±0.061 | 7.366±0.061 | 7.273±0.090 | 7.278±0.080 | 7.283±0.070 |
|  | **60mmHg** | 7.341±0.055 | 7.328±0.055 | 7.279±0.060 | 7.288±0.060 | 7.296±0.060 |
|  | **70mmHg** | 7.303±0.060 | 7.290±0.060 | 7.292±0.056 | 7.311±0.050 | 7.328±0.045 |
|  | **80mmHg** | 7.377±0.052 | 7.361±0.051 | 7.329±0.060 | 7.309±0.060 | 7.291±0.060 |
| **14weeks**  **-female** | **No Treat** | 7.368±0.051 | 7.372±0.048 | 7.249±0.046+ | 7.238±0.046+ | 7.228±0.046 |
|  | **40mmHg** | 7.330±0.046 | 7.334±0.046 | 7.297±0.055 | 7.286±0.055 | 7.276±0.055 |
|  | **50mmHg** | 7.406±0.061 | 7.409±0.061 | 7.335±0.081 | 7.324±0.071 | 7.314±0.062 |
|  | **60mmHg** | 7.368±0.055 | 7.371±0.055 | 7.338±0.060 | 7.321±0.060 | 7.307±0.060 |
|  | **70mmHg** | 7.329±0.061 | 7.332±0.061 | 7.344±0.056 | 7.315±0.044 | 7.289±0.033 |
|  | **80mmHg** | 7.404±0.052 | 7.404±0.052 | 7.381±0.061 | 7.313±0.060 | 7.253±0.059 |
| **14weeks**  **-male** | **No Treat** | 7.401±0.048 | 7.398±0.070 | 7.165±0.046+ | 7.184±0.046+ | 7.201±0.046 |
|  | **40mmHg** | 7.362±0.046 | 7.360±0.046 | 7.212±0.055 | 7.232±0.055 | 7.249±0.055 |
|  | **50mmHg** | 7.439±0.061 | 7.436±0.061 | 7.249±0.074 | 7.269±0.067 | 7.286±0.061 |
|  | **60mmHg** | 7.400±0.056 | 7.397±0.056 | 7.285±0.060 | 7.291±0.060 | 7.295±0.060 |
|  | **70mmHg** | 7.362±0.061 | 7.359±0.061 | 7.371±0.057 | 7.342±0.055 | 7.317±0.053 |
|  | **80mmHg** | 7.437±0.052 | 7.430±0.052 | 7.408±0.061 | 7.340±0.060 | 7.280±0.060 |
| **1.5 year**  **-female** | **No Treat** | 7.325±0.066 | 7.357±0.034 | 7.135±0.046+ | 7.163±0.046+ | 7.186±0.046 |
|  | **40mmHg** | 7.287±0.045 | 7.319±0.045 | 7.183±0.054 | 7.210±0.055 | 7.234±0.055 |
|  | **50mmHg** | 7.363±0.061 | 7.394±0.061 | 7.220±0.039 | 7.247±0.066 | 7.271±0.090 |
|  | **60mmHg** | 7.325±0.055 | 7.356±0.055 | 7.252±0.059 | 7.261±0.060 | 7.269±0.060 |
|  | **70mmHg** | 7.287±0.060 | 7.317±0.060 | 7.329±0.057 | 7.293±0.080 | 7.262±0.100 |
|  | **80mmHg** | 7.361±0.051 | 7.389±0.052 | 7.366±0.060 | 7.291±0.060 | 7.286±0.059 |
| **1.5year**  **-male** | **No Treat** | 7.402±0.048 | 7.332±0.070 | 7.219±0.046 | 7.227±0.046 | 7.234±0.046 |
|  | **40mmHg** | 7.364±0.046 | 7.294±0.045 | 7.267±0.055 | 7.275±0.055 | 7.282±0.055 |
|  | **50mmHg** | 7.440±0.061 | 7.369±0.061 | 7.304±0.075 | 7.312±0.065 | 7.319±0.056 |
|  | **60mmHg** | 7.402±0.056 | 7.331±0.055 | 7.313±0.060 | 7.328±0.060 | 7.341±0.060 |
|  | **70mmHg** | 7.363±0.061 | 7.293±0.060 | 7.335±0.078 | 7.366±0.091 | 7.392±0.103 |
|  | **80mmHg** | 7.438±0.052 | 7.364±0.051 | 7.372±0.060 | 7.363±0.060 | 7.355±0.060 |

Data are mean ±SD (n=8/group); ANOVA analysis showed there were significant differences in the changes of blood pH after fluid infusion between different target resuscitation pressure groups (P<0.05), but no significant difference between ages and sexes.

**Table S2.The changes of PCO_2_(mmHg)**

| **group** | | **baseline** | **End of phase I** | **end of phase II** | **end of phase III** | **end of phase IV** |
| --- | --- | --- | --- | --- | --- | --- |
| **6weeks**  **-female** | **No Treat** | 43.2±6.46 | 35.9±6.37 | 30.9±2.21 | 34.3±2.46 | 37.3±2.67 |
|  | **40mmHg** | 43.0±6.33 | 35.7±5.26 | 29.7±1.29 | 33.0±1.43 | 35.8±1.56 |
|  | **50mmHg** | 45.1±6.63 | 37.5±5.52 | 30.3±2.87 | 33.6±3.03 | 36.6±3.18 |
|  | **60mmHg** | 44.8±6.60 | 37.3±5.49 | 30.7±2.20 | 33.7±2.41 | 36.2±2.59 |
|  | **70mmHg** | 42.9±6.31 | 35.7±5.25 | 31.2±2.91 | 33.7±3.04 | 35.8±3.14 |
|  | **80mmHg** | 45.1±6.63 | 37.5±5.52 | 29.9±2.14 | 33.9±2.72 | 37.4±3.22 |
| **6weeks**  **-male** | **No Treat** | 44.3±7.61 | 38.6±5.22 | 34.7±2.48 | 33.8±2.42 | 33.0±2.36 |
|  | **40mmHg** | 44.0±6.48 | 38.4±5.65 | 33.3±1.45 | 32.4±1.41 | 31.7±1.38 |
|  | **50mmHg** | 46.2±6.80 | 40.3±5.93 | 34.0±3.05 | 33.1±3.01 | 32.3±2.97 |
|  | **60mmHg** | 46.0±6.76 | 40.1±5.90 | 34.5±2.46 | 33.5±2.40 | 32.7±2.34 |
|  | **70mmHg** | 44.0±6.47 | 38.3±5.64 | 35.0±3.10 | 34.0±3.05 | 33.2±3.01 |
|  | **80mmHg** | 46.2±6.80 | 40.3±5.93 | 33.5±2.39 | 34.1±2.76 | 34.6±3.08 |
| **14 weeks**  **-female** | **No Treat** | 45.1±4.32 | 40.4±6.48 | 40.8±2.92 | 41.8±2.99 | 42.8±3.06 |
|  | **40mmHg** | 44.9±6.60 | 40.2±5.92 | 39.1±1.70 | 40.2±1.75 | 41.0±1.79 |
|  | **50mmHg** | 47.1±6.93 | 42.2±6.21 | 39.9±3.35 | 41.0±3.40 | 41.9±3.45 |
|  | **60mmHg** | 46.8±6.89 | 42.0±6.18 | 37.3±2.66 | 40.0±2.86 | 42.3±3.03 |
|  | **70mmHg** | 44.8±6.59 | 40.2±5.91 | 34.3±3.07 | 38.8±3.29 | 42.8±3.49 |
|  | **80mmHg** | 47.0±6.92 | 42.2±6.21 | 32.8±2.35 | 39.1±3.01 | 44.7±3.59 |
| **14weeks**  **-male** | **No Treat** | 42.4±4.18 | 37.8±8.70 | 38.3±2.74 | 38.0±2.72 | 37.8±2.71 |
|  | **40mmHg** | 42.2±6.21 | 37.6±5.54 | 36.7±1.60 | 36.5±1.59 | 36.3±1.58 |
|  | **50mmHg** | 44.3±6.51 | 39.5±5.81 | 37.5±3.23 | 37.3±3.22 | 37.1±3.21 |
|  | **60mmHg** | 44.0±6.48 | 39.3±5.78 | 38.3±2.74 | 37.8±2.70 | 37.3±2.67 |
|  | **70mmHg** | 42.1±6.20 | 37.6±5.53 | 39.3±3.32 | 38.4±3.27 | 37.6±3.23 |
|  | **80mmHg** | 44.2±6.51 | 39.5±5.81 | 37.6±2.69 | 38.4±3.02 | 39.2±3.31 |
| **1.5 year**  **-female** | **No Treat** | 46.3±5.67 | 40.7±1.96 | 43.5±3.54 | 44.9±3.21 | 41.0±2.93 |
|  | **40mmHg** | 46.1±6.78 | 40.5±5.97 | 47.5±2.07 | 43.1±1.88 | 39.3±1.71 |
|  | **50mmHg** | 48.3±7.11 | 42.5±6.26 | 43.4±3.77 | 44.0±3.55 | 40.1±3.36 |
|  | **60mmHg** | 48.1±7.08 | 42.3±6.22 | 42.0±3.50 | 46.0±3.29 | 43.5±3.11 |
|  | **70mmHg** | 46.0±6.77 | 40.4±5.95 | 42.5±3.83 | 44.3±3.77 | 43.2±3.71 |
|  | **80mmHg** | 48.3±7.11 | 42.5±6.26 | 42.4±3.39 | 45.4±3.61 | 45.2±3.81 |
| **1.5 year**  **-male** | **No Treat** | 45.4±4.18 | 38.8±8.64 | 36.8±2.63 | 37.5±2.68 | 38.1±2.73 |
|  | **40mmHg** | 45.2±6.65 | 38.6±5.69 | 35.3±1.54 | 36.0±1.57 | 36.6±1.59 |
|  | **50mmHg** | 47.4±6.97 | 40.5±5.97 | 36.0±3.15 | 36.7±3.19 | 37.4±3.22 |
|  | **60mmHg** | 47.1±6.94 | 40.3±5.93 | 35.8±2.56 | 36.1±2.58 | 36.2±2.59 |
|  | **70mmHg** | 45.1±6.64 | 38.6±5.68 | 35.6±3.13 | 35.3±3.12 | 35.0±3.10 |
|  | **80mmHg** | 47.4±6.97 | 40.5±5.96 | 34.1±2.44 | 35.4±2.83 | 36.6±3.18 |

Data are mean ±SD (n= 8/group); ANOVA analysis showed there is no difference in the changes of partial pressure of carbon dioxide(PCO2) between different target resuscitation pressure groups and different ages and sexes of rats following fluid resuscitation.

**Table S3.The changes of PO_2_(mmHg)**

| **group** | | **baseline** | **End of phase I** | **end of phase II** | **end of phase III** | **end of phase IV** |
| --- | --- | --- | --- | --- | --- | --- |
| **6weeks**  **-female** | **No Treat** | 104.5±8.97 | 100.6±8.66 | 101.3±7.24 | 97.4±6.96 | 94.0±6.72 |
|  | **40mmHg** | 104.0±15.30 | 101.0±17.50 | 144.0±6.26++ | 146.1±6.35** | 147.6±6.43** |
|  | **50mmHg** | 109.1±16.04 | 104.8±18.36 | 147.0±9.91++ | 149.2±10.02** | 151.4±10.11** |
|  | **60mmHg** | 108.5±15.96 | 104.1±18.26 | 146.6±6.38++ | 147.0±6.39** | 147.3±6.41** |
|  | **70mmHg** | 109.8±15.27 | 108.7±17.47 | 146.2±9.87++ | 145.4±9.83** | 144.5±9.79** |
|  | **80mmHg** | 109.0±16.04 | 104.7±18.35 | 139.3±6.06++ | 138.4±6.02** | 137.6±5.99** |
| **6weeks**  **-male** | **No Treat** | 103.8±13.32 | 100.2±17.99 | 100.6±7.19 | 96.7±6.91 | 93.4±6.67 |
|  | **40mmHg** | 103.3±15.19 | 100.5±17.59 | 130.6±5.68++ | 141.6±6.16** | 151.5±6.57** |
|  | **50mmHg** | 108.3±15.94 | 105.4±18.45 | 133.4±9.23++ | 144.5±9.79** | 154.0±10.28** |
|  | **60mmHg** | 107.8±15.85 | 104.7±18.35 | 132.8±5.78++ | 139.6±6.07** | 145.2±6.33** |
|  | **70mmHg** | 103.1±15.16 | 101.3±17.55 | 132.4±9.18++ | 135.8±9.35** | 138.6±9.50** |
|  | **80mmHg** | 108.3±15.93 | 105.4±18.44 | 126.1±5.48+ | 129.3±5.62** | 132.5±5.75** |
| **14 weeks**  **-female** | **No Treat** | 110.7±14.40 | 109.9±11.78 | 102.4±7.32 | 98.5±7.04 | 95.1±6.80 |
|  | **40mmHg** | 105.2±15.47 | 102.3±16.08 | 142.4±6.19++ | 145.4±6.31** | 147.8±6.42** |
|  | **50mmHg** | 110.3±16.23 | 104.6±16.86 | 145.4±9.83++ | 148.2±9.97** | 150.6±10.10** |
|  | **60mmHg** | 109.8±16.15 | 104.0±16.78 | 144.0±6.26++ | 149.4±6.50** | 154.0±6.70** |
|  | **70mmHg** | 105.0±15.45 | 101.1±16.05 | 143.0±9.71++ | 150.2±10.07** | 156.7±10.39** |
|  | **80mmHg** | 110.3±16.23 | 104.6±16.86 | 136.2±5.92++ | 143.4±6.22** | 149.1±6.49** |
| **14weeks**  **-male** | **No Treat** | 105.3±9.88 | 104.3±15.51 | 102.0±7.29 | 98.1±7.01 | 94.7±6.77 |
|  | **40mmHg** | 104.7±15.41 | 103.7±16.72 | 165.3±7.19++ | 172.6±7.51** | 179.6±7.79** |
|  | **50mmHg** | 109.9±16.16 | 109.2±17.54 | 168.8±11.00++ | 176.2±11.37** | 182.9±11.70** |
|  | **60mmHg** | 109.3±16.08 | 108.6±17.45 | 158.3±6.89++ | 161.5±7.02** | 164.7±7.14** |
|  | **70mmHg** | 114.5±15.38 | 103.5±16.69 | 150.3±10.08++ | 150.1±10.07** | 150.2±10.06** |
|  | **80mmHg** | 109.8±16.16 | 106.2±17.54 | 143.2±6.23++ | 143.0±6.22** | 142.8±6.21** |
| **1.5 year**  **-female** | **No Treat** | 105.0±12.58 | 102.8±9.00 | 101.7±7.27 | 97.8±6.99 | 94.4±6.75 |
|  | **40mmHg** | 104.4±15.36 | 102.2±16.50 | 115.8±5.04 | 134.4±5.84** | 140.5±6.55** |
|  | **50mmHg** | 109.5±16.11 | 107.7±17.31 | 118.3±8.48 | 137.2±9.42** | 153.9±10.25** |
|  | **60mmHg** | 109.0±16.03 | 107.1±17.22 | 126.5±5.50+ | 142.6±6.20** | 156.8±6.82** |
|  | **70mmHg** | 108.2±15.33 | 102.0±16.47 | 132.8±9.20+ | 146.8±9.90** | 159.9±10.51** |
|  | **80mmHg** | 109.5±16.11 | 107.6±17.31 | 126.5±5.50+ | 139.8±6.08** | 151.1±6.59** |
| **1.5 year**  **-male** | **No Treat** | 107.4±11.58 | 102.8±9.02 | 104.0±7.44 | 100.1±7.15 | 96.6±6.90 |
|  | **40mmHg** | 106.8±15.72 | 102.2±16.50 | 121.4±5.28 | 122.5±5.33* | 123.0±5.37* |
|  | **50mmHg** | 112.1±16.49 | 107.7±17.31 | 123.9±8.76 | 125.1±8.82* | 126.7±8.87* |
|  | **60mmHg** | 111.5±16.40 | 107.1±17.22 | 124.6±5.42 | 130.6±5.68* | 135.5±5.91* |
|  | **70mmHg** | 106.6±15.69 | 102.0±16.47 | 125.2±8.82 | 134.8±9.30* | 143.0±9.73** |
|  | **80mmHg** | 112.0±16.48 | 107.6±17.31 | 119.2±5.18 | 128.4±5.58* | 136.8±5.93* |

Data are mean ±SD (n= 8/group);PO2: partial pressure of arterial oxygen. ANOVA analysis showed PO2 had significant changes following hemorrhagic shock and fluid infusion between different target resuscitation pressures group(P<0.01), but no significant difference between ages and sexes. ***: *P*<** 0.05, **P<0.01 vs no treatment group.+P<0.05, ++P<0.01 as compared to at the end of phase I.
