# Supplementary material for: Ideal resuscitation pressure for uncontrolled hemorrhagic shock in different ages and sexes of rats
Source: Crit Care. 2013 Sep 10;17(5):R194. doi: 10.1186/cc12888 (PMC4264615; doi:10.1186/cc12888)
Supplement: Additional file 7 — is Figure S1 showing effects of different target resuscitation pressures on liver and renal function in different ages and sexes of rats after uncontrolled hemorrhagic shock. Data are mean ± standard deviation (n= 8/group). (A) changes in alanine aminotransferase (ALT) levels; (B) changes in aspartate aminotransferase (AST) level; (C) changes in blood urea nitrogen (BUN); (D) changes in serum creatinine (Scr). Analysis of variance showed these parameters had significant changes following hemorrhagic shock and fluid infusion between ages and different target resuscitation pressures(P < 0.01), but no significant difference between sexes (P > 0.05). #P < 0.05, ##P < 0.01 versus 40 mmHg group. [file cc12888-S7.pptx]

## Slide 1
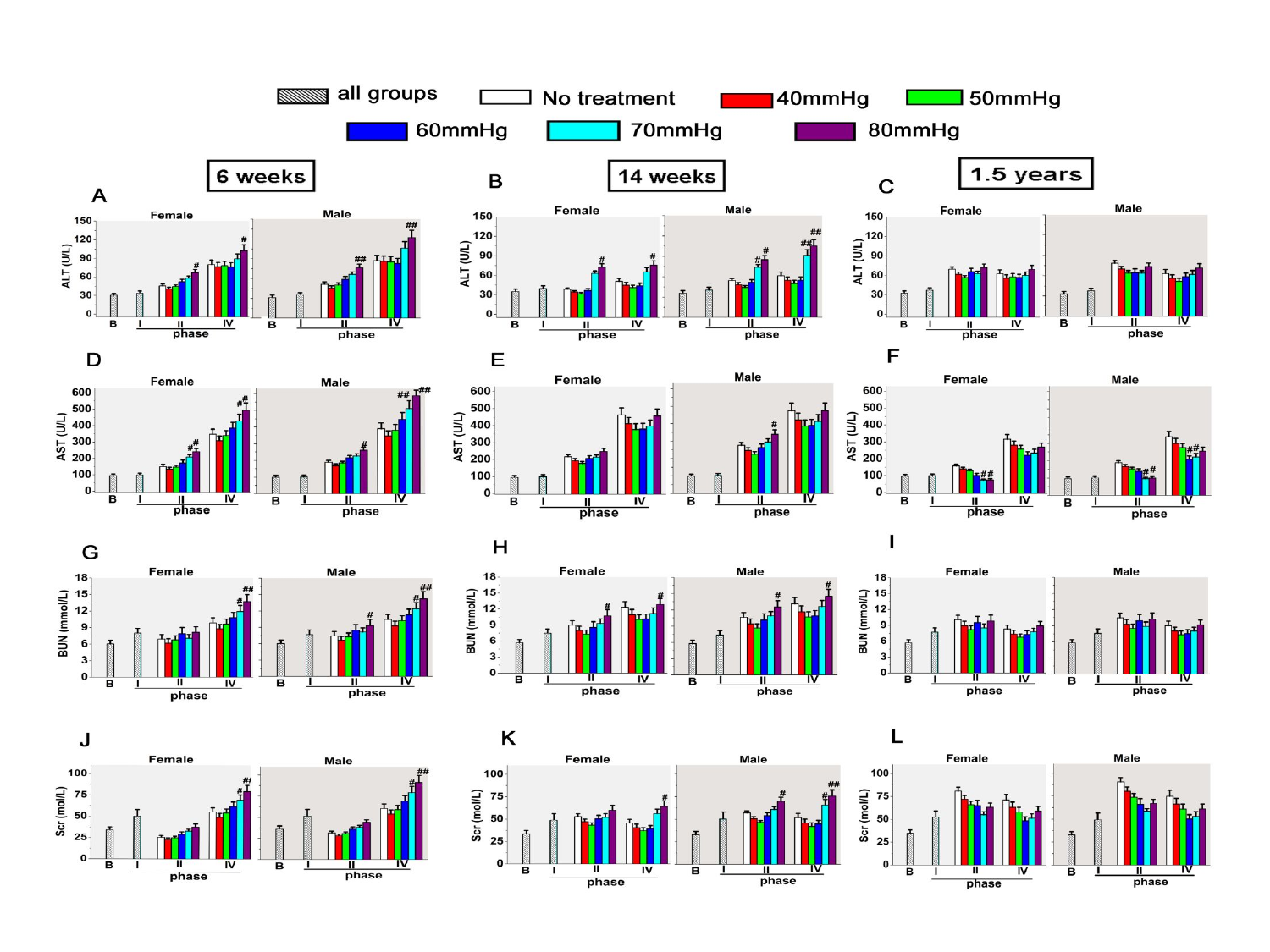

## Slide 2
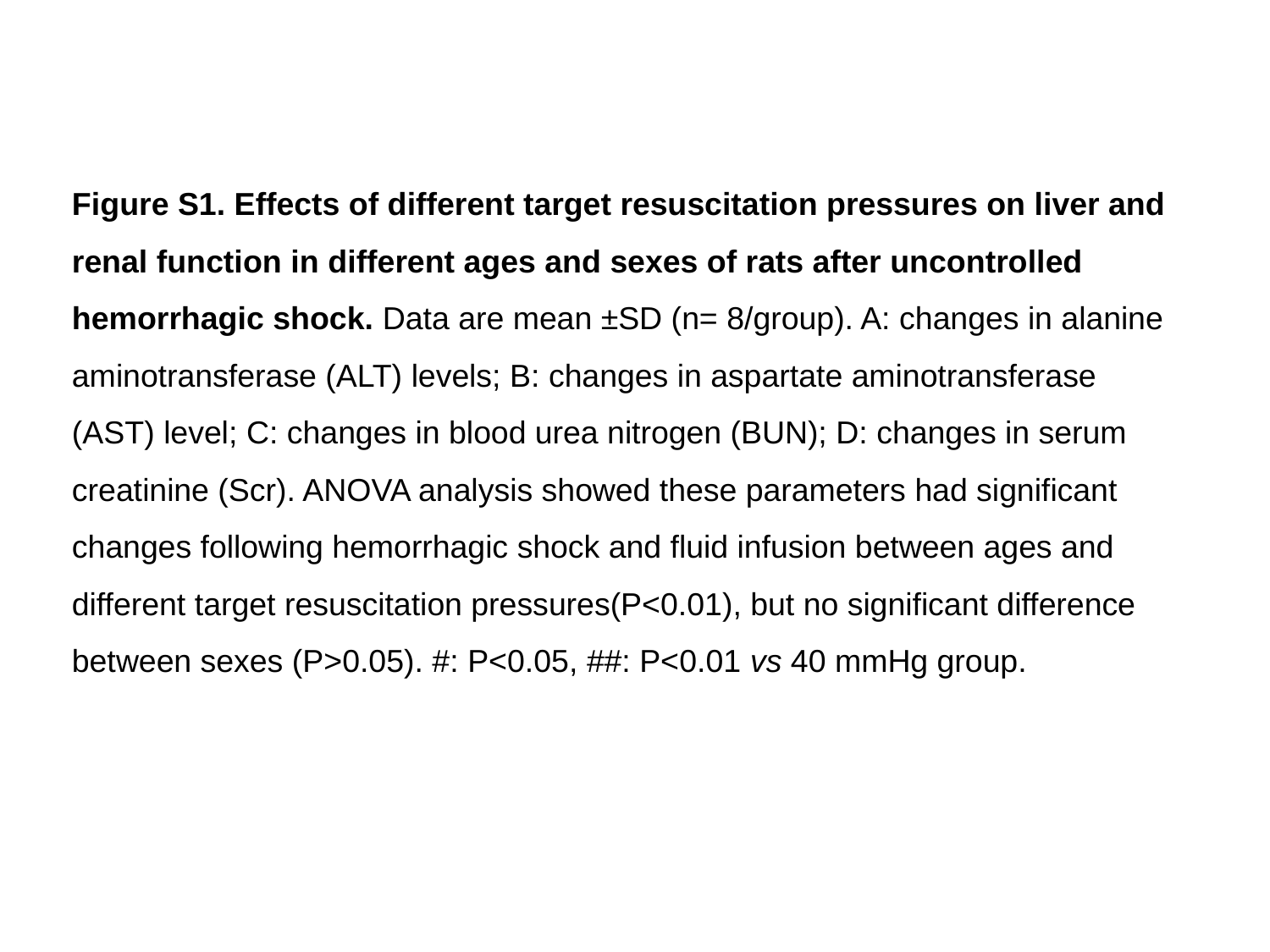

Figure S1. Effects of different target resuscitation pressures on liver and renal function in different ages and sexes of rats after uncontrolled hemorrhagic shock. Data are mean ±SD (n= 8/group). A: changes in alanine aminotransferase (ALT) levels; B: changes in aspartate aminotransferase (AST) level; C: changes in blood urea nitrogen (BUN); D: changes in serum creatinine (Scr). ANOVA analysis showed these parameters had significant changes following hemorrhagic shock and fluid infusion between ages and different target resuscitation pressures(P<0.01), but no significant difference between sexes (P>0.05). #: P<0.05, ##: P<0.01 vs 40 mmHg group.
