# Supplementary material for: Ideal resuscitation pressure for uncontrolled hemorrhagic shock in different ages and sexes of rats
Source: Crit Care. 2013 Sep 10;17(5):R194. doi: 10.1186/cc12888 (PMC4264615; doi:10.1186/cc12888)
Supplement: Additional file 8 — is Table S4 presenting the effects of different pressures on coagulation parameters. [file cc12888-S8.docx]

**Table S4. Effects of different pressures on coagulation parameters**

| **Group** | | | | **Baseline** | | **End of Phase I** | | **end of phaseII** | | **end of phase III** | | **2h of phase IV** | |
| --- | --- | --- | --- | --- | --- | --- | --- | --- | --- | --- | --- | --- | --- |
| **PT-INR** | | | | | | | | | | | | | |
| **6weeks** | | **Female-50** | | **1.08±0.13** | | **0.97±0.11** | | **1.15±0.10** | | **1.26±0.11** | | **1.48±0.12*** | |
|  |  | **Female-70** | |  |  |  |  | **1.14±0.10** | | **1.25±0.12** | | **1.49±0.11*** | |
|  |  | **Male-50** | | **1.07±0.10** | | **0.99±0.10** | | **1.16±0.10** | | **1.28±0.10 *** | | **1.52±0.14*** | |
|  |  | **Male-70** | |  |  |  |  | **1.15±0.04** | | **1.36±0.11*** | | **1.63±0.13*** | |
| **14weeks** | | **Female-50** | | **1.08±0.19** | | **1.08±0.14** | | **1.16±0.10** | | **1.28±0.09** | | **1.45±0.12*** | |
|  |  | **Female-70** | |  |  |  |  | **1.17±0.10** | | **1.27±0.08** | | **1.46±0.15*** | |
|  |  | **Male-50** | | **1.19±0.15** | | **1.17±0.36** | | **1.29±0.13** | | **1.27±0.14** | | **1.38±0.16** | |
|  |  | **Male-70** | |  |  |  |  | **1.25±0.14** | | **1.20±0.13** | | **1.47±0.12** | |
| **1.5years** | | **Female-50** | | **1.15±0.12** | | **1.03±0.16** | | **1.23±0.11** | | **1.29±0.11** | | **1.34±0.12** | |
|  |  | **Female-70** | |  |  |  |  | **1.22±0.11** | | **1.30±0.09** | | **1.33±0.11** | |
|  |  | **Male-50** | | **1.26±0.20** | | **1.16±0.22** | | **1.36±0.12** | | **1.35±0.12** | | **1.38±0.12** | |
|  |  | **Male-70** | |  |  |  |  | **1.38±0.12** | | **1.34±0.11** | | **1.37±0.11** | |
| **APTT（s）** | | | | | | | | | | | | | |
| **6weeks** | | **Female-50** | | **26.4±1.62** | | **27.7±3.22** | | **32.3±3.35*** | | **38.9±3.90**** | | **56.8±5.39**** | |
|  |  | **Female-70** | |  |  |  |  | **35.8±3.64*** | | **42.5±4.20**** | | **62.5±5.86**** | |
|  |  | **Male-50** | | **25.7±2.31** | | **25.8±3.71** | | **33.8±3.47*** | | **40.5±4.03**** | | **55.9±5.32**** | |
|  |  | **Male-70** | |  |  |  |  | **36.5±3.70*** | | **44.9±4.40**** | | **61.9±5.82**** | |
| **14weeks** | | **Female-50** | | **27.3±6.90** | | **30.0±13.63** | | **32.5±5.86** | | **42.8±6.72**** | | **58.5±8.03**** | |
|  |  | **Female-70** | |  |  |  |  | **38.9±6.40** | | **49.8±7.31**** | | **62.5±8.36**** | |
|  |  | **Male-50** | | **27.7±5.49** | | **35.2±18.10** | | **41.5±5.78*** | | **51.5±6.61**** | | **57.4±7.94**** | |
|  |  | **Male-70** | |  |  |  |  | **39.5±6.45*** | | **50.0±7.32**** | | **58.5±8.03**** | |
| **1.5 years** | | **Female-50** | | **31.4±3.47** | | **33.0±7.07** | | **40.5±4.03*** | | **50.8±4.89**** | | **60.5±5.70**** | |
|  |  | **Female-70** | |  |  |  |  | **41.2±4.09*** | | **51.9±5.56**** | | **66.5±6.20**** | |
|  |  | **Male-50** | | **32.8±5.22** | | **29.9±8.70** | | **41.9±4.15*** | | **49.8±4.81**** | | **61.2±5.75**** | |
|  |  | **Male-70** | |  |  |  |  | **43.6±4.29*** | | **56.4±5.35**** | | **64.3±6.01**** | |
| **PT (s)** | | | | | | | | | | | | | |
| **6weeks** | | **Female-50** | | **13.7±2.01** | | **14.0±1.63** | | **18.9±1.85*** | | **26.5±2.54**** | | **25.6±2.45**** | |
|  |  | **Female-70** | |  |  |  |  | **20.1±2.87*** | | **28.5±2.72**** | | **26.8±2.56**** | |
|  |  | **Male-50** | | **13.5±1.61** | | **14.2±1.48** | | **19.5±1.90*** | | **28.6±2.63**** | | **28.5±2.53**** | |
|  |  | **Male-70** | |  |  |  |  | **21.5±2.08*** | | **29.6±2.81**** | | **29.1±2.59**** | |
| **14weeks** | | **Female-50** | | **14.6±2.88** | | **14.6±2.22** | | **19.8±1.92*** | | **27.9±2.66**** | | **28.5±2.72**** | |
|  |  | **Female-70** | |  |  |  |  | **21.3±2.06*** | | **30.1±2.86**** | | **29.5±2.81**** | |
|  |  | **Male-50** | | **14.5±2.41** | | **15.6±5.98** | | **22.8±3.11*** | | **28.9±3.66**** | | **33.5±3.53**** | |
|  |  | **Male-70** | |  |  |  |  | **23.6±3.18*** | | **30.2±3.78**** | | **34.6±3.63**** | |
| **1.5 years** | | **Female-50** | | **13.6±1.83** | | **14.9±2.52** | | **21.5±2.08*** | | **30.2±2.87**** | | **32.8 ±3.11**** | |
|  |  | **Female-70** | |  |  |  |  | **25.6±2.45*** | | **31.5±2.99**** | | **33.0±3.12**** | |
|  |  | **Male-50** | | **14.5±3.12** | | **15.8±3.38** | | **22.3±2.15*** | | **31.2±2.96**** | | **31.0±2.95**** | |
|  |  | **Male-70** | |  |  |  |  | **26.5±2.53*** | | **32.5±3.08**** | | **32.0±3.03**** | |
| **FIB (g/L)** | | | | | | | | | | | | |  |
| **6weeks** | | **Female-50** | | **1.10±0.27** | | **1.18±0.32** | | **1.11±0.18** | | **1.12±0.17** | | **1.10±0.17** |  |
|  |  | **Female-70** | |  |  |  |  | **1.12±0.18** | | **1.11±0.17** | | **1.01±0.16** |  |
|  |  | **Male-50** | | **1.13±0.58** | | **1.17±0.43** | | **1.22±0.20** | | **1.15±0.20** | | **1.08±0.19** |  |
|  |  | **Male-70** | |  |  |  |  | **1.19±0.20** | | **1.12±0.19** | | **1.05±0.18** |  |
| **14weeks** | | **Female-50** | | **1.28±0.20** | | **1.09±0.06** | | **1.01±0.15** | | **0.98±0.15** | | **0.95±0.14** |  |
|  |  | **Female-70** | |  |  |  |  | **1.02±0.15** | | **0.97±0.14** | | **0.96±0.11** |  |
|  |  | **Male-50** | | **1.26±0.23** | | **1.21±0.12** | | **1.12±0.16** | | **1.02±0.15** | | **1.01±0.15** |  |
|  |  | **Male-70** | |  |  |  |  | **1.09±0.15** | | **0.99±0.13** | | **1.03±0.12** |  |
| **1.5years** | | **Female-50** | | **1.17±0.20** | | **1.07±0.24** | | **1.02±0.15** | | **0.98±0.15** | | **0.97±0.14** |  |
|  |  | **Female-70** | |  |  |  |  | **1.03±0.15** | | **0.97±0.14** | | **0.98±0.15** |  |
|  |  | **Male-50** | | **1.14±0.15** | | **1.12±0.30** | | **1.08±0.20** | | **0.99±0.19** | | **0.97±0.17** |  |
|  |  | **Male-70** | |  |  |  |  | **1.04±0.19** | | **0.98±0.16** | | **0.96±0.12** |  |
| **TT (s)** | | | | | | | | | | | | |  |
| **6weeks** | | **Female-50** | | **23.3±1.84** | | **25.5±5.19** | | **32.8±2.86*** | | **38.9±3.37**** | | **52.6±4.51**** |  |
|  |  | **Female-70** | |  |  |  |  | **33.6±2.92*** | | **40.2±3.48**** | | **54.6±4.67**** |  |
|  |  | **Male-50** | | **22.4±1.34** | | **26.8±3.70** | | **31.2±2.73*** | | **40.2±3.32**** | | **51.2±4.39**** |  |
|  |  | **Male-70** | |  |  |  |  | **32.9±2.87*** | | **39.6±3.42**** | | **50.1 ±4.30**** |  |
| **14weeks** | | **Female-50** | | **21.0±7.53** | | **26.3±7.45** | | **33.2±2.89*** | | **39.8±3.44**** | | **50.2±4.31**** |  |
|  |  | **Female-70** | |  |  |  |  | **35.9±3.12*** | | **38.1±3.30**** | | **54.8±4.69**** |  |
|  |  | **Male-50** | | **20.4±3.18** | | **24.7±6.07** | | **33.0±2.87*** | | **42.3±3.65**** | | **54.2 ±4.64**** |  |
|  |  | **Male-70** | |  |  |  |  | **34.6±3.01*** | | **43.2±3.73**** | | **55.0±4.70**** |  |
| **1.5years** | | **Female-50** | | **25.6±0.89** | | **29.1±5.47** | | **32.8±2.86*** | | **37.8±3.28**** | | **51.0 ±4.37**** |  |
|  |  | **Female-70** | |  |  |  |  | **33.9±2.95*** | | **38.2±3.31**** | | **52.6±4.51**** |  |
|  |  | **Male-50** | | **25.9±4.93** | | **29.0±5.70** | | **31.0±2.71*** | | **42.1±3.63**** | | **49.5±4.25**** |  |
|  |  | **Male-70** | |  |  |  |  | **32.1±2.80*** | | **44.1±3.80**** | | **50.2±4.31**** |  |

Data are mean ±SD (n= 8/group); PT-INR: international normalized ratio of prothrombin time; PT: prothrombin time; TT: thrombin time; APTT: activated partial prothrombin time; FIB: fibrinogen. ANOVA analysis showed these parameters had no significant changes following hemorrhagic shock and fluid infusion between ages, sexes and different target resuscitation pressures group(P>0.05).*P<0.05, **P<0.01 was as compared with baseline in a same resuscitation pressure group.
